# Evaluating the ICD-11 PTSD and Complex PTSD Constructs: A Meta-Analytic Confirmatory Factor Analysis of the International Trauma Questionnaire

**DOI:** 10.1177/10731911251340837

**Published:** 2025-05-30

**Authors:** Reuben Kindred, Suzanne Jak, Ruby Hamer, Maja Nedeljkovic, Glen W. Bates

**Affiliations:** 1 Swinburne University of Technology, Melbourne, VIC, Australia; 2 University of Amsterdam, The Netherlands

**Keywords:** International Trauma Questionnaire, PTSD, complex post-traumatic stress disorder, trauma, factor analysis, ICD-11, meta-analysis

## Abstract

The International Trauma Questionnaire (ITQ) is a widely used diagnostic tool for post-traumatic stress disorder (PTSD) and complex PTSD (CPTSD). Although findings are mixed, investigations of the ITQ’s internal structure have most often supported a six-factor first-order model and a two-factor second-order model. This study utilized meta-analytic factor analysis to investigate the ITQ’s latent structure. A systematic search of PsycINFO, Web of Science, PubMed, MEDLINE, Cochrane, Scopus, CINAHL, and ProQuest Dissertations was conducted, with 57 studies (total *N* = 43,066) included in the final analysis. A two-stage meta-analytic structural equation modeling approach was implemented which pooled correlation matrices and performed confirmatory factor analyses. The two-factor second-order model demonstrated good fit, emphasizing its clinical relevance. However, a seven-factor correlated first-order model demonstrated superior fit, consistently outperforming other models across ITQ versions, symptom severity levels, English versus translated versions, and both PTSD and non-PTSD samples. This model indicates that CPTSD encompasses a broad range of symptoms and specifically that the distinction between affective hyperactivation and hypoactivation is important to progress the construct validity of CPTSD. Additionally, reliability analyses revealed limited internal consistency at the subscale level. These findings highlight the need for comprehensive assessments and refined item content to enhance CPTSD measurement.

The International Trauma Questionnaire (ITQ) is an 18-item self-report measure developed to assess post-traumatic stress disorder (PTSD) and complex PTSD (CPTSD). CPTSD was incorporated into the 11th iteration of the International Classification of Diseases (ICD-11) released by the World Health Organization in 2018. The ITQ was designed in accordance with the ICD-11 principle that disorders should focus on a limited but central set of symptoms (Christen et al., 2021). The ITQ has been translated into 25 languages and validated for use in both clinical and non-clinical populations. Studies exploring the underlying structure of the ITQ have yielded mixed findings concerning the fundamental dimensionality of the ITQ (Redican et al., 2021), highlighting the need for further investigation of its factorial validity. Since the ICD-11 serves as the global classification system for mental health disorders, and the ITQ is the only self-report measure tailored to assess these diagnoses, it is important to establish the factorial validity of the ITQ as a measure of ICD-11 PTSD and CPTSD.

Several earlier versions of the ITQ were developed, including both a 22-item and 28-item ITQ. Using item response theory, several items in these preliminary versions of the ITQ were rejected due to low thresholds for endorsement. An abbreviated measure (henceforth, ITQ-12) was officially released in 2018 (Cloitre et al., 2018), consisting of 12 items relating to PTSD and CPTSD symptomatology, along with 6 functional impairment items. The ITQ-12 asks participants to answer items in relation to a specific traumatic event and produces scores in six symptom clusters under the core PTSD and disturbances in self-organization (DSO) second-order factors. The core PTSD second-order factor comprises three two-item symptom clusters: *re-experiencing the event*, *avoidance of internal and external reminders of the event*, and *a sense of threat* (e.g., being watchful or on guard). The DSO factor also comprises three two-item symptom clusters: *affective dysregulation* (e.g., exaggerated emotional reactivity), *negative self-concept* (e.g., feeling like a failure), and *disturbances in relationships* (e.g., feeling distant from others). In addition to the two second-order factors, functional impairment questions ask whether the PTSD and DSO symptoms have affected their functioning in daily life. These items do not count towards the major subscales but are used for categorical scoring for a diagnosis of PTSD and CPTSD. CPTSD is diagnosed if the criteria for both PTSD and DSO are met, along with their respective functional impairment. Psychometric investigations of the ITQ have provided good evidence of its internal consistency of the total ITQ-12 (e.g., α = .84; Gerrmann et al., 2024), superordinate factors (e.g., α = .76–.83; Vallières et al., 2018), and subscales (e.g., α = .67–.90; Ho et al., 2019). Research has also established the construct validity of the ITQ (Hyland et al., 2017; Karatzias et al., 2016; Shevlin et al., 2018)—including criterion-related validity (Hansen et al., 2021) and both convergent and discriminant validity (Cyr et al., 2022; Gerrmann et al., 2024; Hyland et al., 2019).

Several studies have explored the factor structure of CPTSD with a recent qualitative review by Redican et al. (2021) suggesting that the correlated six-factor models and the two-factor second-order model are the most supported structures in the factorial analyses of the ITQ-12 and its preliminary versions. Specifically, a two-factor second-order model—consistent with the ICD-11 PTSD and CPTSD conceptualization—was found to be the best-fitting model in clinical samples; whereas, the correlated six-factor first-order model was the best-fitting model in most community samples. The authors argued that the lower prevalence rate in the community may have contributed to the lack of delineation in the community samples. The variability in which model performs better across different samples and contexts has prompted further investigation into the factorial structure of the ITQ (Reed et al., 2022).

Alternative structural models have also received empirical support. Previous factor analyses have indicated a total of nine different factor models of the ITQ as producing acceptable fit. For example, a correlated seven-factor model that separates the affective dysregulation symptom cluster into two dimensions of hyperactivation and hypoactivation was deemed the best fitting in several studies using the preliminary versions of the ITQ (Ben-Ezra et al., 2018; Vallières et al., 2018). Separating this symptom cluster has been argued to enhance the construct validity of the DSO subscale and allow for more targeted symptom reduction strategies (Karatzias et al., 2018). Clarifying the ITQ’s factor structure is crucial for accurate diagnosis, effective interventions, and improved patient outcomes in PTSD and CPTSD. To date, there has been no quantitative synthesis examining the latent structure of the ITQ.

In the present study, a meta-analytic confirmatory factor analysis was conducted to investigate the dimensionality of the ITQ. Our study had three primary aims. First, we aimed to identify the best-fitting factor structure for the ITQ on the combined data. First, we identified the best-fitting factor structure by comparing nine ITQ-12 models from previous studies and assessing these models in preliminary and modified ITQ versions. Second, as factor structure may vary depending on the characteristics of the sample, we sought to examine potential moderating variables of the model fit—assuming that statistical heterogeneity was present in the main analysis. To achieve this aim, a moderator analysis was conducted using variables relating to sample type (i.e., PTSD samples vs. non-PTSD samples, samples that met CPTSD threshold vs. those that did not, and English vs. translated versions of the ITQ). Calculating reliability coefficients allows for a deeper evaluation of how well each factor model fits the data, providing crucial insights into the consistency of the constructs being measured, and ultimately enhancing the validity of comparisons between models (Scherer & Teo, 2020). As such, a third aim was to estimate the reliability coefficients of the best-fitting model and, additionally, the model proposed by the ICD-11 for PTSD and CPTSD, as this model is widely used in clinical practice and could potentially be the best-fitting model. Relatedly, we looked to assess whether the internal consistency of the ITQ varies significantly between different groups based on sample type, as above.

## Method

### Protocol and Registration

The current systematic review adhered to the Preferred Reporting Items for Systematic Reviews and Meta-Analyses (PRISMA) guidelines and was registered with PROSPERO (ID: CRD42023434942). The PRISMA checklist is in Supplemental Table 1.

### Identification and Selection of Studies

A systematic search was conducted on April 1, 2023 and April 22, 2024. Relevant studies were identified in PsycINFO, Web of Science, PubMed, MEDLINE, Cochrane, Scopus, CINAHL, and ProQuest Dissertations and Theses. The ITQ was formally released in 2018, but development began in 2015, so a date restriction from 2015 was applied. No language restrictions were used. The primary focus was on cross-sectional studies, but clinical trials and experimental studies were included if ITQ data were collected pre-manipulation. Longitudinal studies with baseline covariances for multiple ITQ administrations were also included.

The keywords searched were: “CPTSD” OR “C-ptsd” OR “Complex PTSD” OR “Complex Post traumatic stress” OR “complex trauma disorder” OR “disturbances in self-organization” OR “International Trauma Questionnaire.” Medical Subject Headings do not yet exist for CPTSD, so these were not implemented. Records were imported into EndNote X9, duplicates were removed, and two authors (R.K. and R.H.) screened titles and abstracts. Inter-rater reliability for title and abstract screening was Cohen’s Kappa = .91, prior to discrepancies being resolved by discussion. Full texts were obtained for eligible studies. Details of the search strategy are in Supplemental Table 2.

To be included, studies were required to have (a) administered any version of the ITQ and (b) provided a covariance matrix and item level mean scores—either as part of the published document or provided this data to us upon request via email. When original publications did not include appropriate inter-item matrices, the authors were contacted by email. If the corresponding author did not respond to an initial email, a follow-up email was sent 4 weeks later. Studies were excluded if they were a study protocol, the full texts were unavailable, or the covariance matrix was non-positive definite.

**Figure. 1.**
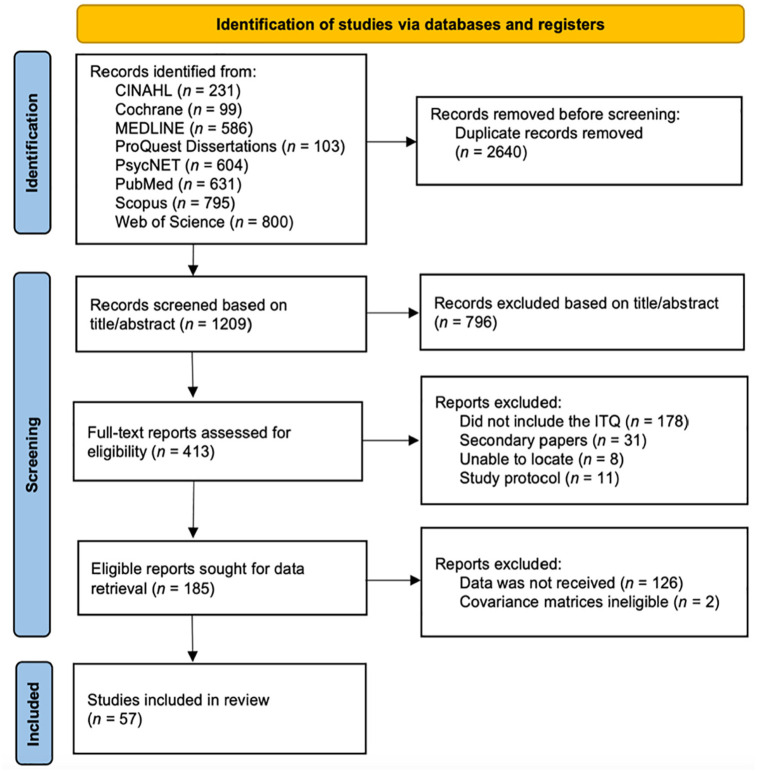
PRISMA Flowchart of Study Selection Process. *Note.* PRISMA = Preferred Reporting Items for Systematic Reviews and Meta-Analyses.

Full-text publications were independently screened for eligibility by R.K. and R.H. with inter-rater reliability at Cohen’s Kappa = .93. Disagreements were resolved by discussion. However, none of the studies provided a full covariance matrix in their published document. Therefore, we emailed 126 authors to obtain data relating to 185 studies (author response *k* = 48, 38%). Details of the search strategy and identification are supplied in Figure 1.

### Coding Procedure and Data Extraction

Data fields were extracted using a data extraction template in Microsoft Office Excel. The included studies were coded on study-level and measure characteristics. These included: study author/s, study publication year, sample size, country, age of sample, gender demographics, translation used, and whether the translation was back-translated. Data extraction was conducted by R.K. and R.H., discrepancies were identified in 8 out of 472 data points, resulting in an accuracy rate of 98.3%. Discrepancies were resolved through discussion. Studies were deemed to have a PTSD-diagnosed sample if most participants were identified as having a diagnosis of PTSD or CPTSD through a clinical interview, such as the Clinician-Administered PTSD Scale for DSM-5 (Weathers et al., 2018), or if the participation requirements in the study specified an official trauma-related diagnosis. A probable CPTSD classification was estimated based on ITQ scoring guidelines as a proxy for diagnosis rather than a formal diagnostic category. To qualify for this classification, studies needed to have item means of ≥2 in at least one of two items from each of the six symptom clusters: re-experiencing in the here and now, avoidance, sense of current threat, affective dysregulation, negative self-concept, and disturbances in relationships (ITQ; Cloitre et al., 2018). Overall severity on the ITQ was also calculated for each study. This criterion provides an approximation of CPTSD symptom burden at the sample level.

### Study Quality Assessment

A modified version of the Quality Assessment Tool for Quantitative Studies (QualSyst; Kmet et al., 2004) was employed to establish study quality based on four criteria: selection bias, disclosure of study’s participants, data collection methods, and data quality. For each criterion, studies were coded as on a three-point scale (1 = *strong*, 2 = *moderate*, 3 = *weak*). To establish a comprehensive quality rating for each study, the average of the four criteria ratings was calculated. Assessment of study quality was conducted by R.K. and R.H. independently. Cohen’s Kappa was calculated at .89, indicating strong agreement between coders (Landis & Koch, 1977). Any discrepancies were resolved through discussion.

### Statistical Analysis

#### Measures

The ITQ (Cloitre et al., 2018) is a self-administered questionnaire consisting of 12 items split across PTSD and DSO subscales along with six functional impairment items. Items are rated on a 5-point Likert-type scale (0 = *not at all*; 4 = *extremely*). Both subscales consist of a functional impairment factor (e.g., “In the past month, have the above problems affected your work or ability to work?”). These were excluded from the current analysis as they do not relate to the dimensionality of CPTSD. There was sufficient adequate data to synthesize the preliminary 22-item version of the ITQ, but not enough data to analyze the 28-item ITQ. Three modified model versions were employed to explore why certain latent structure models outperformed others and evaluate the robustness of the measure’s latent structure across different item configurations.

Overlapping items can lead to artificially high inter-item correlations, which may distort the factor structure and inflate fit indices. By removing proposed redundant items (Frewen et al., 2023), a 20-item ITQ aimed to ensure that each item contributed unique variance to the constructs being measured. Factors represented by a single item lack sufficient variance to reliably measure a construct. As such, two 14-item versions of the ITQ—incorporating the 12 items from the finalized version and 2 from the preliminary versions—allowed the examination of split affective dysregulation factors without relying on latent factors represented by a single item. Supplemental Table 3 contains the items in these ITQ versions.

#### Factors Examined

**Figure 2.**
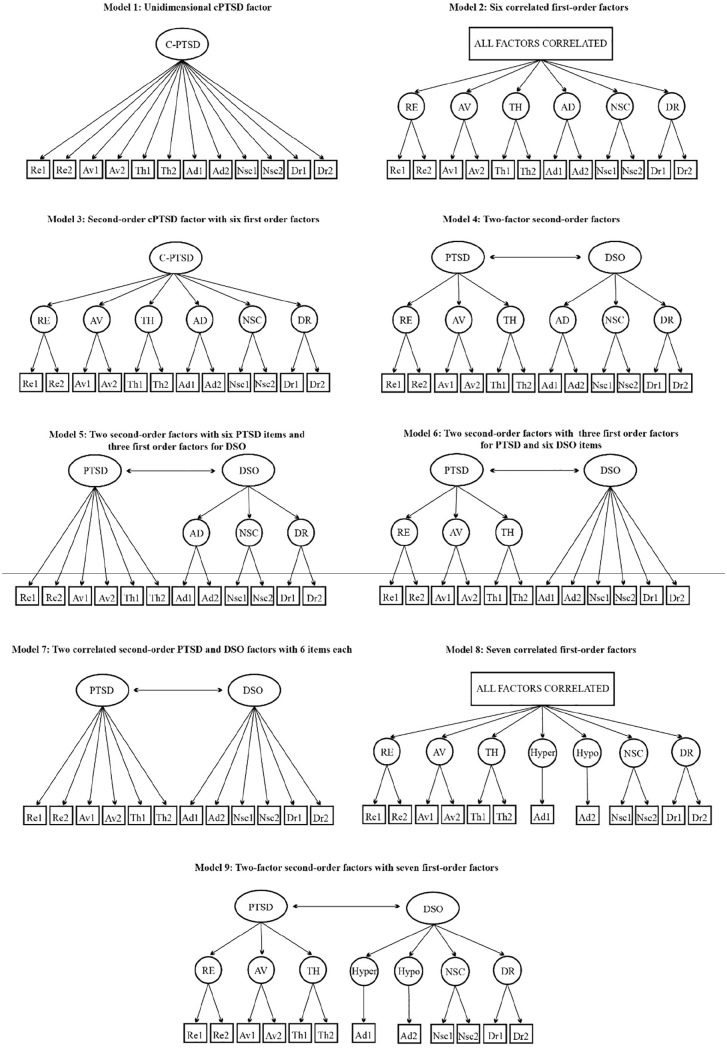
Factor Models for the Finalized ITQ-12. *Note*. ITQ = International Trauma Questionnaire.

We ran the analysis on the nine-factor structures suggested in the literature, including lower-order and higher-order models. Figure 2 shows item loadings for each model on the ITQ-12, while Supplemental Figure 1 presents those for the ITQ-22.

#### Non-Positive Definite Intercorrelation Matrix

The meta-analytic structural equation modeling approach assumes that the covariance matrices are positive definite, meaning that they have no negative eigenvalues (Oh, 2020). A non-positive definite matrix violates this assumption and can lead to estimation problems and biased results, which can invalidate the conclusions drawn from the analysis (Wothke, 1993). Two studies (i.e., Maercker et al., 2018; Niwa et al., 2022) with a non-positive definite covariance matrix were excluded prior to analysis as per recommendations (Jak, 2015).

#### Two-Stage Structural Equation Modeling Approach

The factor structure of the ITQ was determined by analyzing correlation matrices using a random-effects two-stage structural equation modeling (TSSEM) approach (Cheung & Chan, 2005). In the first stage of a TSSEM analysis, the diagonal elements of each collected covariance matrix were set to one and the off-diagonal elements were standardized to convert the matrix into a correlation matrix. A multivariate meta-analysis was then applied to synthesize these matrices. For random-effects analyses, the Cochran’s *Q* statistic and the Higgins *I*^2^ statistic were used to assess heterogeneity. The Cochran’s *Q* chi-squared test was employed to assess the significance of variability in study effect sizes not attributable to chance. A statistically significant *Q* rejects the null hypothesis of homogeneity. The Higgins *I*^2^ statistic was computed, which represents the percentage of between-studies variance in the observed effect sizes. A conventional interpretation of Higgins *I*^2^ is that 25% signifies low, 50% denotes moderate, and 75% indicates high heterogeneity (Borenstein et al., 2009). In the second stage of TSSEM, weighted least squares estimation (Browne, 1984) is used to fit structural equation models on the pooled correlation matrix determined in the first stage. Specifically, the weight matrix is the inverse of the estimated asymptotic variance–covariance matrix of the pooled correlation coefficients. We identified the scale of the common factors by fixing their variances to 1. To identify the single-indicator factors (in Models 8 and 9), in addition, the residual indicator variance was fixed at zero. This ensures that the single-indicator factors accurately reflect their respective indicators.

Model fit was assessed with the *χ*^2^ statistic and six goodness of fit indices: the standardized root mean square residual (SRMR), the root mean square error of approximation (RMSEA), the comparative fit index (CFI), the Tucker–Lewis index (TLI), and, to aid model comparison, the Akaike Information Criterion (AIC) and Bayesian Information Criterion (BIC). Lower values of RMSEA and SRMR suggest a more favorable fit, with values below 0.08 considered acceptable and values below 0.05 as good fit (Hu & Bentler, 1999). For CFI and TLI, we considered values exceeding 0.90 as indicative of an acceptable fit, while those above 0.95 are representative of a good fit (Hu & Bentler, 1999). The AIC and BIC lack direct interpretability on their own, but lower values indicate a better trade-off between goodness of fit and model complexity, with models having lower or negative BIC values being preferable over those with higher, positive scores (Burnham & Anderson, 2004).

To assess the robustness of our findings, three sensitivity analyses were conducted: (a) excluding low and moderate overall quality studies, (b) excluding studies that were not back-translated, and (c) excluding studies with low missingness quality criteria.

#### Moderator Analyses

Moderator hypotheses relating to the model fit were investigated with three moderators relating to sample characteristics. To do this, a subgroup analysis was conducted by pooling the correlation coefficients of each subgroup and comparing the fit of the factor models across the separate groups (Jak & Cheung, 2018). Three subgroup analyses were conducted: one comparing PTSD versus non-PTSD subgroups, one comparing subgroups that reached probable CPTSD thresholds with those that did not, and one comparing translated versus English versions of the ITQ. To reduce the risk of Type I errors, only models with good fit were assessed, these were then compared with goodness of fit indices previously described. In these analyses, it was planned to use random-effects models; however, there were not enough studies in each group to be able to fit a random effects model in each subgroup analysis.

Therefore, a one-stage meta-analytic structural equation modeling (Jak & Cheung, 2020) was used to conduct a moderator analysis on the parameter estimates of the Stage 2 model. This allowed for random-effects modeling in the total group of studies, ensuring the reliability of statistical inferences. A significant omnibus χ^2^ test (*p* ≤ .05) indicated moderation by subgroup variables, including PTSD versus non-PTSD, probable CPTSD versus non-CPTSD, and translated versus English ITQ versions. While probable CPTSD was categorized based on diagnostic scoring, overall symptom severity was treated as a continuous variable and standardized for numerical stability.

#### Reliability Analyses

Reliability coefficients were estimated based on the best-fitting model in the second stage of the analysis (see Scherer & Teo, 2020). Typically, model preference should be based solely on model fit, with the reliability of the observed scale assessed subsequently for the selected model. However, we considered it important to conduct the reliability analysis on the ICD-11 model, even if it was not the best-fitting model. This is because the ICD-11 model is the globally accepted standard for diagnosing PTSD and CPTSD, and its second-order factors are well supported by empirical research and substantively meaningful. By analyzing the reliability of the ITQ within this framework, the study contributes to validating the existing standards.

The analysis involved the computation of composite reliability using coefficient omega, or ω (McDonald, 1999) and omega second-order, or ω_ho_ (Zinbarg et al., 2006), using matrix algebra expressions that delineate the proportion of variance attributable to the common factor for each respective subscale. Coefficients between 0.70 and 0.79 were considered acceptable, between 0.80 and 0.89 were considered good, and ≥0.90 were considered excellent (Cicchetti, 1994).

#### Statistical Software and Data Availability

All statistical analyses were conducted with version 4.0.3 of R (R Core Team, 2018), using the metaSEM (version 1.3.1; Cheung, 2015), OpenMx (version 2.20.6; Neale et al., 2016), and lavaan (version 0.6.15; Rosseel, 2012) packages. The data and scripts used in this review can be accessed at OSF website https://osf.io/nbpvx/

## Results

### Selection and Characteristics of Studies

The present review included data from 31 different countries with studies being published between 2016 and 2024. One originated from unpublished data sources (i.e., Kindred, unpublished data). The median sample size was 307 participants (mean = 743; range: 44–4,944). The mean age of participants in studies ranged from 19.09 to 67.08 (median = 37.4). The percentage of women in studies ranged from 6.20% to 100% (mean: 62%). The majority of studies (*k* = 50) administered the ITQ released in 2018, although several studies had implemented the preliminary versions of the ITQ (*k* = 8). Eighteen studies conducted the ITQ exclusively in its English version. The majority of studies (*k* = 39, percentage = 68.4%) used translated versions of the ITQ in one or more of the following languages: Arabic, Simplified Chinese, Traditional Chinese, Danish, Dari, Dutch, French, German, Hebrew, Japanese, Korean, Lithuanian, Norwegian, Polish, Russian, Rwandan, Slovenian, Spanish, and Turkish. Of the studies using translated versions, 36 (92.3%) had undergone back-translation.

Study characteristics are supplied in Table 1. In total, data from 43,066 relevant participants were synthesized across 57 studies with 58 unique samples obtained. Each correlation coefficient was calculated with 55 to 58 samples and sample sizes for each correlation coefficient ranged between 37,921 and 43,066. The number of studies and total sample sizes for each correlation coefficient for both the ITQ-12 and ITQ-22 are in Supplemental Tables 4 to 6. Based on our criteria, 9 studies utilized a PTSD sample and 48 a non-PTSD sample. Based on item means, 16 studies exhibited endorsement patterns suggestive of CPTSD cut-off scores (serving as a proxy for probable CPTSD), while 41 studies did not meet this threshold. Item means for the entire sample and each sub-sample are in Supplemental Tables 7 and 8.

**Table 1:** Summary of Included Studies.

Study	Country	Sample type	*N*	Mean age (*SD*)	Gender (female %)	ITQ measure	Clinical interview	Probable CPTSD	Quality assessment
Alpay and Aydın (2024)	Turkey	Earthquake survivors	261	29.20 (28.06)	62.0%	ITQ-12^a,b^	No	No	Strong
Andisha et al. (2023)	Austria	Asylum seekers and refugees	305	31.38 (11.05)	33.8%	ITQ-12^a,b^	No	No	Strong
Armour et al. (2021)	Ireland	Veterans	732	55.88 (10.59)	9.70%	ITQ-12	No	No	Strong
Astill Wright et al. (2021)	UK	Primary and secondary health care service users	222	49 (13)	49.1%	ITQ-12	No	Yes	Strong
Bækkelund et al. (2022)	Norway	Clinical sample (outpatients)	186	37.3 (12.1)	67.20%	ITQ-12^a,b,c^	Existing PTSD required	Yes	Strong
Barbieri et al. (2022)	Niger	Treatment-seeking asylum-seekers	126	26.12 (6.88)	24.60%	ITQ-12^a^	No	Yes	Strong
Ben-Ezra et al. (2018)	Israel	Representative national community sample	1,003	40.6 (14.5)	51.70%	ITQ-22^a,b,c^	No	No	Strong
Ben-Ezra et al. (2020)	Kenya, Nigeria, and Ghana	Representative national community sample	2,524	Group average: 30.45 (7.93–9.36)	49.56%	ITQ-12	No	No	Strong
Bongaerts et al. (2022)	The Netherlands	Treatment-seeking clinical sample	44	37.16 (11.82)	50.70%	ITQ-12^a,b^	CAPS-5	Yes	Strong
Brewin et al. (2022)	UK	Trauma-exposed police officers	2,021	35–44	42%	ITQ-12	No	Yes	Strong
Camden et al. (2023)	USA	University students	246	19.8 (4.62)	82.50%	ITQ-12	No	No	Strong
Charak et al. (2023)	Spain	LGBTQ+ community	225	31.35 (9.48)	28.8%	ITQ-12^a,b^	No	No	Strong
Chiu et al. (2023)	Taiwan	Community sample	119	26.35 (3.43)	56%	ITQ-12^a,b^	No	No	Strong
Choi, Kim, and Lee (2021)	Korea	Organized violence survivors	236	67.08 (10.93)	19.50%	ITQ-12^a,b^	No	No	Strong
Choi, Lee, and Hyland (2021)	Korea	Community sample	800	40.74 (10.92)	48.75%	ITQ-12^a,b^	No	No	Strong
Dighton et al. (2023)	UK	Veterans	2,185	30–39	7.05%	ITQ-12	No	No	Moderate
Engle (2019)	USA	Community sample	94	35.74 (14.29)	58%	ITQ-12	No	No	Strong
Engle (2022)	USA	Community sample	62	32.2 (12.15)	69%	ITQ-12	No	Yes	Moderate
Folke et al. (2021)	Denmark	Treatment-seeking veterans	599	39.9 (9.7)	6.20%	ITQ-12^a,b^	No	No	Strong
Fung et al. (2023)	China	Primary healthcare service users	376	40.48 (12.59)	80.90%	ITQ-12^a,b^	No	No	Moderate
Gerrmann et al. (2024)	The Netherlands	Treatment seeking clinical sample	4,944 (ITQ-12) and 956 (ITQ-28)	41 (12.8)	70.60%	ITQ-12 + ITQ-28^a,b^	CAPS-5	Yes	Strong
Greenblatt-Kimron et al. (2023)	Israel	Community sample	603	41.65 (13.8)	68.66%	ITQ-12^a,b^	No	No	Strong
Greene et al. (2023)	UK	Health and social care workers	1,056	41.7 (0.2)	92.6%	ITQ-12	No	Yes	Strong
Gündoğmuş et al. (2023)	Turkey	Clinical and community sample	395	32.43 (10.14)	61.00%	ITQ-12^a,b^	No	No	Moderate
Ho et al. (2020)	Multi-sample	University students	1,346	20 (1.55)	67.90%	ITQ-12^a,b^	No	No	Strong
Ho et al. (2021)	Ireland	Community sample	1,020	43.1 (15.12)	51.00%	ITQ-12	No	No	Strong
Huang et al. (2023)	China	Community sample	1,361	20.65 (1.85)	67.6 %	ITQ-12^a,b^	No	No	Strong
Kairyte et al. (2022)	Lithuania	Community sample	158	33.61 (9.73)	85.40%	ITQ-12^a,b^	No	Yes^d^	Strong
Kampling et al. (2022)	Germany	Community sample	2,004	51.3 (18.1)	52.50%	ITQ-12^a,b^	No	No	Strong
Kindred et al. (unpublished)	Australia	Community sample	408	29.9 (11)	72.50%	ITQ-22^c^	No	No	N/A
Knefel et al. (2016)	Austria	Foster care childhood abuse survivors	219	57.95 (9.54)	40.20%	ITQ-22^a,b^	No	No	Strong
Knefel et al. (2019)	Multi-sample	Community sample	1,591	43.55 (15.1)	67.70%	ITQ-12^a,b^	No	No	Strong
Knefel et al. (2022)	Austria	Treatment seeking asylum seekers and refugees	88	34.3 (13.6)	49%	ITQ-12^a,b^	No	No	Strong
Kuhar and Kocjan (2022)	Slovenia	Community sample	4,847	46.7 (15.3)	49.10%	ITQ-28^a,c^	No	No	Strong
Li et al. (2024)	China	College bullying victims	675	19.6 (1.34)	65.2%	ITQ-12^a^	No	No	Strong
Liddell et al. (2019)	Australia	Treatment seeking refugees	112	37.7 (11.5)	33.9%	ITQ-28^c^	PSS-I	No	Moderate
Masters (2023)	USA	Community sample	48	Group average: 25–34	100%	ITQ-12	No	No	Moderate
Mutuyimana and Maercker (2022)	Rwanda	Genocide survivors	261	46.3 (11.95)	52.90%	ITQ-12^a,b^	No	No	Strong
Padun et al. (2022)	Russia	Community sample	429	37.5 (10.7)	74%	ITQ-12^a,b^	No	No	Strong
Panayi et al. (2022)	UK	Mental health service users with psychosis	144	40.43 (13.82)	42.40%	ITQ-12	No	Yes	Strong
Peraud et al. (2022)	France	Community sample	750	34.4 (12.9)	89.20%	ITQ-12^a,b^	No	No	Strong
Rentmeesters and Hermans (2023)	Belgium	Police officers	1,476	40.68 (9.62)	28.90%	ITQ-12^a,b^	No	No	Moderate
Rink and Lipinska (2020)	South Africa	University students	576	20.46 (2.76)	84.55%	ITQ-12	No	No	Strong
Rzeszutek et al. (2021)	Poland	Adult children of alcoholics	609	35.48 (9.77)	85.70%	ITQ-12^a,b^	No	Yes	Moderate
Sandberg and Refrea (2022)	USA	University students	169	19.27 (2.4)	74%	ITQ-12	No	No	Strong
Sele et al. (2020)	Norway	Treatment-seeking clinical sample	201	41.5 (9.5)	84.70%	ITQ-22^a,b,c^	Existing PTSD required	Yes	Strong
Simon et al. (2019)	UK	Clinical sample	199	47.36 (12.57)	50%	ITQ-12	CAPS-5	Yes	Moderate
Triantafyllou (2023)	USA	Community sample	167	22.30 (2.33)	91.60%	ITQ-12	No	No	Moderate
Truskauskaite et al. (2023)	Lithuania	University students	1,626	19.09 (1.05)	68.20%	ITQ-12^a,b^	No	No	Strong
Tsur (2020)	Israel	Community sample—daughters	194	26 (3.03)	100%	ITQ-12^a,b^	No	No	Strong
Tsur (2020)	Israel	Community sample—mothers	194	56 (6.3)	100%	ITQ-12^a,b^	No	No	Strong
Tsur and Abu-Raiya (2020)	Israel	Community sample	837	47.69 (14.02)	82.90%	ITQ-12^a,b^	No	No	Strong
Valdovinos et al. (2023)	Mexico	Treatment-seeking clinical sample	112	46.13 (9.8)	100%	ITQ-12^a,b^	Existing PTSD required	Yes^d^	Strong
Vallières et al. (2018)	Lebanon	Treatment-seeking refugees	110	33.02 (8.94)	80.20%	ITQ-22^a,b^	No	Yes	Strong
Voorendonk et al. (2020)	Netherlands	Treatment-seeking clinical sample	308	41.26 (12.7)	77.60%	ITQ-12^a,b^	CAPS-5	Yes	Moderate
Webb et al. (2022)	UK	Clinical sample (inpatient)	45	25.5 (6.81)	100%	ITQ-12	Existing EUPD Required	Yes	Moderate
Zerach and Levi-Belz (2022)	Israel	Health and social care workers	299	40.28 (10.83)	77.60%	ITQ-12^a,b^	No	No	Strong
Zerach and Levi-Belz (2023)	Israel	Female veterans	1,119	26.03 (4.72)	100%	ITQ-12^a,b^	No	No	Strong

*Note.* CAPS-5 = Clinician-Administered PTSD Scale for DSM-5; CPTSD = complex PTSD; DSO = disturbances in self-organization; EUPD = emotionally unstable personality disorder; ICD = International Classification of Diseases; ITQ = International Trauma Questionnaire; PTSD = post-traumatic stress disorder; PSS-I = PTSD Symptom Scale–Interview version; N/A = not applicable.

a Administered translated version/s.

b Confirmed to implement a back-translated version of the ITQ.

c Select items only.

d Item means in one of the symptom cluster was very close (<.13) to reaching the threshold; therefore, we coded this as probable CPTSD.

### Methodological Quality

Overall study quality was rated as moderate to high. The majority of studies, representing 79% (*k* = 45), were considered high quality based on the QualSyst (Kmet et al., 2004) study quality coefficients. Twelve studies (21%) were categorized as moderate in quality and none was assessed as low quality. For selection bias, nearly all studies (94.6%, *k* = 53) were rated as high quality, reflecting appropriate participant selection, while only three studies (5.4%) were rated as moderate. In the domain of disclosure of participants, all studies (*k* = 56) were rated as high quality, with comprehensive reporting of participant demographics, ensuring transparency, and reproducibility. For data collection methods, 75% of studies (*k* = 42) were rated as strong, although 13 studies (23.2%) were rated as moderate due to incomplete reporting of ITQ reliability. One study (1.8%) was rated as weak because of unclear data collection procedures. The data quality criterion highlighted the most significant limitation, with nearly half of the studies (*k* = 27; 48.2%) rated as weak, primarily due to a failure to report missing data (*k* = 24). Five studies (8.9%) were rated as moderate, and 24 studies (42.9%) were rated as strong. Overall study quality is indicated in Table 1, and quality scores for each criterion can be found in Supplemental Table 9.

### Meta-Analytic Confirmatory Factor Analysis

In the first stage of the main analysis using the random-effects model, heterogeneity was detected in the data for the ITQ-12, with *Q* = 18,230.74 (degrees of freedom = 3,586, *p* < .001). The *I*^2^ statistic revealed a large range in the heterogeneity of correlations across studies (*I*^2^ range = 11.15–97.49%). Supplemental Table 10 provides the pooled correlation matrix of Stage 1 under the random effects model and *I*^2^ index at the item level.

The evaluation of each of the nine factor models involved an examination of fit indices and standardized factor loadings on the hypothesized factors. The unidimensional model clearly exhibited an unsatisfactory fit (e.g., CFI = 0.87, SRMR = 0.12). In contrast, most multidimensional models showed at least acceptable fits. For instance, the TLI and CFI fit statistics were within acceptable limits for Models 2, 4, 5, 6, 8, and 9. Models showing good fit included the correlated six-factor models (Model 2) and the two-factor second-order model (Model 4), and their counterparts which included a split affective dysregulation factor (Models 8 and 9).

The seven-factor model (i.e., Model 8) was ultimately selected as the final model based on the relative fit indices. In this model, every item showed positive and substantial factor loadings on their respective first-order factors, with standardized values ranging from .75 to .92. There were also notable correlations between all first-order factors, ranging from .41 to .90 for Model 2 and .42 to .77 for Model 8. Specific factor correlations of the models are displayed in Supplemental Tables 11 and 12. The sensitivity analyses confirmed the robustness of the results. Model 8 was superior when excluding low and moderate quality studies, non-back-translated studies, and studies with low missingness quality criteria. Results of sensitivity analyses are presented in Supplemental Tables 13 to 15.

**Figure 3.**
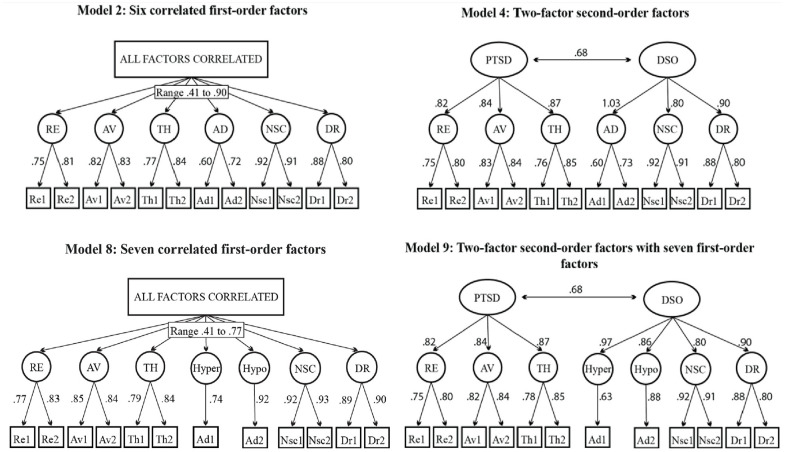
Standardized Factor Loadings for Models Reaching Good Fit for the ITQ-12. *Note*. ITQ = International Trauma Questionnaire.

It is important to consider the results of the two-factor second-order model, as it represents the ICD-11 structure for PTSD and CPTSD, has a large amount of empirical support, and is prominent in existing literature. The two-factor second-order model (i.e., Model 4) was designated the third best-fitting model, based on relative fit indices. All items loaded onto the first-order factors significantly (range: 0.60–0.92), and first-order factors loaded onto high-order factors significantly (range: 0.80–1.03). The factor correlation coefficient between core PTSD (i.e., ICD-11 PTSD) and DSO (i.e., a superordinate factor of CPTSD) was *r* = .68, which implies that the two constructs were moderately related but are also distinguishable factors with unique characteristics. Fit indices for all models are presented in Table 2 and parameter estimates of good-fitting models in Figure 3.

**Table 2:** Model Fit Statistics.

Model	χ^2^	*df*	RMSEA	SRMR	TLI	CFI	AIC	BIC
Random-effects analysis run on all studies (*k* = 57; *N* = 43,066)								
**1**	7,285.24	54	0.056	0.123	0.840	0.869	7,177.24	6,709.04
**2**	449.63	39	0.016	0.020	0.987	0.993	371.63	33.48
**3**	4,159.07	48	0.044	0.086	0.898	0.926	4,063.07	3,646.89
**4**	830.29	47	0.020	0.029	0.980	0.986	736.29	328.77
**5**	1,431.04	50	0.025	0.046	0.967	0.975	1,331.04	897.51
**6**	1,948.28	50	0.030	0.049	0.955	0.966	1,848.28	1,414.76
**7**	2,548.89	53	0.033	0.061	0.943	0.955	2,442.89	1,983.35
**8**	**215.16**	**33**	**0.011**	**0.012**	**0.993**	**0.997**	**149.16**	**−136.97**
**9**	836.82	46	0.020	0.029	0.980	0.986	744.82	345.97

*Note.* Values in bold indicate the best-fitting models in each category. AIC = Akaike Information Criterion; BIC = Bayesian Information Criterion; CFI = comparative fit index; *df* = degrees of freedom; RMSEA = root mean square error of approximation; SRMR = standardized root mean square residual; TLI = Tucker–Lewis Index.

## Moderator Analyses of Model Comparison

Models 2, 4, 8, and 9, identified as having good fit, were tested across multiple moderators. Model 8 consistently demonstrated the best fit across all six subsamples, followed by Model 2. These first-order models outperformed others across PTSD and non-PTSD samples, probable CPTSD and non-CPTSD groups, and studies administering the ITQ in either English or a translated version. Results are presented in Table 3.

**Table 3:** Fit Statistics for Good Fitting Models by Subgroup.

Model	*χ* ^2^	*df*	RMSEA	SRMR	TLI	CFI	AIC	BIC
Fixed-effects analysis run on studies with PTSD samples (*k* = 9; *N* = 6,151*)*								
** 2**	485.78	39	0.043	0.033	0.994	0.996	407.78	145.53
** 4**	645.24	47	0.046	0.043	0.993	0.995	551.24	235.20
** 8**	**307.77**	**33**	**0.037**	**0.022**	**0.996**	**0.998**	**241.77**	**19.86**
** 9**	647.58	46	0.046	0.043	0.993	0.995	555.58	246.26
Random-effects analysis run on studies with non-PTSD samples (*k* = 48; *N* = 36,915*)*								
** 2**	381.09	39	0.016	0.020	0.989	0.994	303.01	−28.85
** 4**	734.11	47	0.020	0.029	0.982	0.987	640.11	240.08
** 8**	**186.88**	**33**	**0.011**	**0.012**	**0.994**	**0.997**	**120.88**	−**159.99**
** 9**	739.15	46	0.020	0.029	0.981	0.987	647.15	255.64
Fixed-effects analysis run on studies that met the complex PTSD threshold (*k* = 16; *N* = 10,547)								
** 2**	748.88	39	0.043	0.034	0.993	0.996	670.88	391.04
** 4**	985.78	47	0.046	0.043	0.993	0.995	891.78	554.53
** 8**	**424.59**	**33**	**0.035**	**0.020**	**0.996**	**0.998**	**358.59**	**121.80**
** 9**	992.51	46	0.046	0.044	0.993	0.995	900.51	570.43
Random-effects analysis run on studies that did not meet complex PTSD threshold (*k* = 41; *N* = 32,519)								
** 2**	310.96	39	0.015	0.018	0.992	0.995	232.96	−95.17
** 4**	675.37	47	0.020	0.028	0.984	0.988	581.37	185.92
** 8**	**157.31**	**33**	**0.011**	**0.012**	**0.995**	**0.998**	**91.31**	−**186.34**
** 9**	677.10	46	0.020	0.028	0.983	0.988	585.10	198.07
Fixed-effects analysis run on studies with studies using translated versions (*k* = 37; *N* = 29,319)								
** 2**	1,269.37	39	0.033	0.025	0.995	0.997	1,191.37	868.22
** 4**	2,040.41	47	0.038	0.042	0.994	0.996	1,946.41	1,556.97
** 8**	**821.49**	**33**	**0.029**	**0.015**	**0.997**	**0.998**	**755.49**	**482.05**
** 9**	2,053.92	46	0.039	0.042	0.994	0.996	1,961.92	1,580.77
Fixed-effects analysis run on studies with studies using English (*k* = 19; *N* = 12,030)								
** 2**	799.42	39	0.040	0.030	0.991	0.995	721.42	433.01
** 4**	1,211.47	47	0.045	0.046	0.989	0.992	1,117.47	769.90
** 8**	**587.15**	**33**	**0.037**	**0.023**	**0.993**	**0.996**	**521.15**	**277.11**
** 9**	1,212.56	46	0.046	0.046	0.989	0.992	1,120.56	780.39

*Note.* Values in bold indicate the best-fitting model in each category. AIC = Akaike Information Criterion; BIC = Bayesian Information Criterion; CFI = comparative fit index; *df* = degrees of freedom; RMSEA = root mean square error of approximation; SRMR = standardized root mean square residual; TLI = Tucker–Lewis Index; PTSD = post-traumatic stress disorder.

### Secondary Analysis of Long-Form and Modified Versions of the ITQ

When conducting the analysis on the ITQ-22, data from 7,856 relevant participants were synthesized across eight studies. Studies were classified as PTSD (*k* = 5), non-PTSD (*k* = 3), reaching probable CPTSD thresholds (*k* = 3), and under probable CPTSD thresholds (*k* = 5). When conducting the analysis on the ITQ-22, a fixed-effects model was used due to low number of studies. The chi-square test for homogeneity of correlation coefficients was significant (χ^2^(1,111) = 3,128.94, *p* < .001), indicating that exact homogeneity of the correlation coefficients across studies is rejected. However, the correlation matrix derived from the fixed effects analysis could be utilized to fit the structural model. Supplemental Table 16 provides the pooled correlation matrix of Stage 1 under the fixed-effects model. Fit statistics were within acceptable limits for all models. Acceptable fit was found for Models 2 and 4. Most goodness of fit indices indicated good fit for Models 8 and 9. Using relative fit indices, the seven correlated first-order factor model (i.e., Model 8) demonstrated superior fit.

Interestingly, in the two-factor second-order model (i.e., Model 4), the factor correlation coefficient between PTSD and DSO was *r* = .86, suggesting a substantial overlap in the variance captured by PTSD and DSO factors. These results need to be interpreted with caution due to the small number of studies. Refer to Supplemental Table 17 for results of the fit indices. Parameter estimates of the seven correlated first-order factor model and the two-factor second-order model of the ITQ-22 are displayed in Supplemental Figure 2. There were also notable correlations between all first-order factors, ranging from .59 to .89 for Model 8, and .59 to .88 for Model 2. Specific factor correlations of the models are displayed in Supplemental Tables 18 and 19.

Three modified ITQ versions were used to control for measurement concerns and remove potentially redundant items, as these may impact the factor structure results. Using relative fit indices, all revised versions of the ITQ revealed that the seven correlated first-order factor model exhibited superior fit—refer to Supplemental Table 20 for results. The findings indicate that the factorial structure of the long-form version of the ITQ was likely not an artifact of redundant items or that singleton factors artificially inflated fit indices in the factorial analysis of the ITQ-12. Taken together, the superiority of the seven correlated first-order factor model (i.e., Model 8) replicated across the ITQ-12, ITQ-22, and modified versions of the ITQ.

### Reliability Analyses

For the ITQ-12, the results show that the best-fitting model’s (Model 8) reliability varies from unacceptable to good, depending on the subscale. While the ITQ-22 improves reliability in some aspects, several subscales still fail to meet acceptable reliability thresholds. The ICD-11 model (Model 4) demonstrated reliable internal consistency. Table 4 displays the reliability coefficients that were estimated using TSSEM for the best-fitting model (Model 8) and for the ICD-11 model (i.e., Model 4) across the ITQ-12 and ITQ-22.

**Table 4:** Composite Reliability Estimates by Measure and Model.

Model	Factor	ITQ-12	ITQ-22
Model 2/Model 8	Re	ω = .61 (.58–.64)	ω = .66 (.63–.68)
	Av	ω = .69 (.67–.72)	ω = .77 (.74–.79)
	Th	ω = .66 (.64–.69)	ω = .74 (.71–.76)
	Ad	ω = .43 (.41–.46)	ω = .55 (.55–.56)
	Hyper	ω = N/A	ω = .49 (.48–.50)
	Hypo	ω = N/A	ω = .66 (.65–.67)
	Nsc	ω = .84 (.82–.85)	ω = .82 (.80–.82)
	Dr	ω = .71 (.69–.72)	ω = .78 (.76–.79)
Model 4	Core PTSD	ω_ho_ = .84 (.83–.84)	ω_ho_ = .89 (.88–.89)
	DSO	ω_ho_ = .87 (.86–.87)	ω_ho_ = .96 (.96–.96)

*Note.* Ad = affect dysregulation; Av = avoidance; Dr = disturbances in relationships; DSO = disturbances in self-organization; Hyper = hyperactivation; Hypo = hypoactivation; N/A = not applicable; Nsc = negative self-concept; PTSD = post-traumatic stress disorder; Re = re-experiencing; Th = sense of threat; ITQ = International Trauma Questionnaire.

That the reliability estimates of the ITQ-12 did not reach acceptable thresholds when using the first-order model are, in part, due to having only two items per subscale. Unacceptable reliability is found in the core PTSD subscales (i.e., Re-experiencing, Avoidance, Sense of Threat) and the Affective Dysregulation subscale. The ITQ-22, which has more items for three of its subscales, generally produced higher subscale reliability scores, except for the Negative Self-Concept subscale. For both the ITQ-12 and ITQ-22, the Affective Dysregulation subscale had lower reliability compared to other subscales. When the Affective Dysregulation scale is represented by two subscales, as in the seven correlated first-order factor model (Model 8), analyses using ITQ-22 data revealed that the Affective Hyperactivation subscale demonstrated lower reliability, while the Affective Hypoactivation subscale showed higher reliability. However, neither subscale reached acceptable reliability thresholds. The likelihood of this being a consequence of the measure’s item count is minimal, given that the Affective Hyperactivation and Hypoactivation subscales contain five and four items, respectively.

Due to the good fit of the two-factor second-order model, theoretical basis, and diagnostic usage, assessing the reliability of the ITQ within the widely used ICD-11 framework, offers comparative insights, and provides guidance for future research and application across diverse contexts. The outcomes of the reliability analysis reveal that the two-factor second-order model (i.e., Model 4) exhibited good to excellent internal consistency for the ITQ-12 and ITQ-22.

#### Moderator Effect on Factor Loadings and Reliability Estimates

In several subgroups, it was not possible to conduct random-effects analyses. To address this, a meta-regression approach was applied, enabling the identification of systematic variations in factor loadings across levels of several moderators. Absolute differences smaller than .10 in factor loadings were considered insubstantial (Kaplan, 1989), indicating limited evidence of meaningful moderation or measurement non-invariance. The omnibus test revealed a significant effect of the PTSD sample moderator on all factor loadings, χ^2^(12) = 61.80, *p* < .001. The effect was negative for each factor loading, and the mean change in factor loadings was Δλ = −.10, which is negligible. Similarly, for studies meeting probable CPTSD thresholds, the factor loadings were significantly lower than the other studies, χ^2^(12) = 585.73, *p* < .001. The mean change in factor loadings was Δλ = −.11 in these studies, which is bordering on negligible. Total symptom severity significantly moderated factor loadings, *χ*^2^(12) = 61.80, *p* < .001, with both negative and positive changes ranging from −0.04 to 0.01 and the mean change remained negligible at Δλ = −.01. Language moderation effects were significant as well, χ^2^(12) = 62.38, *p* < .001. The mean change in factor loadings for studies administering the ITQ in English was negligible at Δλ = −.01, with negative and positive changes ranging from −.06 to .06. These results indicate that first-order models are stable, and while moderation effects exist, they do not substantially alter the factor loadings. The individual moderating effects for each moderator on the factor loadings in the first-order model are presented in Supplemental Table 21.

Given the statistically significant moderation of factor loadings, an analysis was performed to assess whether the internal consistency of the ITQ-12 varied significantly between different sample types, evaluating both the best-fitting model (Model 8) and the two-factor second-order model (Model 4). Inspection of the regression coefficients of the moderating effects indicates that the factor loadings were significantly lower in the studies with PTSD samples and in those reaching a probable CPTSD diagnostic threshold. This implies lower reliability in those groups of studies. Because of the statistically significant moderator analyses, reliability coefficients were estimated within these separate subgroups.

Studies with PTSD samples exhibited lower reliability estimates compared to those with non-PTSD samples, with differences in reliability ranging from Δω = −.06 in the Sense of Threat and Negative Self-Concept subscales to Δω = −.15 in the Re-experiencing subscale. Generally, lower reliability estimates were observed in groups with probable CPTSD, compared to samples not meeting this criterion, including a notable decrease of Δω = −.22 in Re-experiencing subscale. Conversely, the Negative Self-Concept subscale demonstrated higher estimates, with an increase of Δω = .05. For translated studies, reliability was consistent across subgroups, except for increases in Δω = .06 in the Sense of threat and a decrease of Δω = −.10 in Affective Dysregulation subscales.

However, using second-order models (i.e., Models 4 and 9), all subgroups displayed acceptable to good internal consistency at the factor level (e.g., DSO). As first-order models indicated lower reliability, this indicates that the superior fit of first-order models likely reflects a more precise representation of the factor structure rather than differences in measurement precision across model types. Reliability estimates for subgroups can be found in Supplemental Table 22.

## Discussion

In this meta-analytic confirmatory factor analysis, our study had three primary aims: (a) to identify the best-fitting factor model for the ITQ, (b) to examine potential moderating variables affecting model fit, and (c) to estimate the reliability coefficients for both the best-fitting model and the ICD-11 model. In total, 57 studies contributed data on the ITQ’s latent structure.

This discussion first considers the best-fitting model for the ITQ, the seven correlated first-order factor model in terms of its theoretical implications, including the differentiation between affective hyperactivation and hypoactivation. The superiority of this model is confirmed across various samples, contrasting past research. We then discuss the ICD-11 model and propose several reasons why it did not reach superiority. Next, we discuss reliability considerations. One drawback of first-order factor models is their limited reliability estimates at the subscale level, even when using the longer-form version of the ITQ. Taken together, there is a need for further refinement of the ICD-11 PTSD and CPTSD concepts and that, due to insufficient coverage of complex symptomatology by the ICD-11 diagnostic criteria, comprehensive assessments for ICD-11 PTSD and CPTSD are warranted.

### Model Fit for the ITQ

The seven correlated first-order factor model (Ben-Ezra et al., 2018) demonstrated a considerably better fit compared to alternative factor models. Superiority of this model was also replicated across modified and preliminary long-form versions of the ITQ. These results suggest that the symptoms of CPTSD can be considered as distinct and relatively independent constructs. This aligns with the idea that CPTSD encompasses a broad range of symptoms that go beyond the core symptoms of PTSD and emphasizes the heterogeneity of experiences among individuals with CPTSD. This model is consistent with a dimensional approach (Wolf et al., 2015) and the symptom-based network model of PTSD (Knefel et al., 2019)—where symptom clusters interact with each other without implying an underlying disease entity.

Within the finalized ITQ and ICD-11 diagnostic criteria, affective hyperactivation and hypoactivation are considered as two congeneric measures of a single underlying dimension. Our findings suggest that replacing a general affective dysregulation factor with separate affective hyperactivation and affective hypoactivation subscales resulted in improved model fit, in the main analysis, in each examined measure, and in all subsamples. Redican et al. (2021) also noted that, albeit with limited factorial analyses, improved fit in models that separate affective dysregulation into two independent factors (e.g., the seven correlated first-order model). Likewise, the results are consistent with findings from network analysis studies, which indicate that these two symptoms are not strongly associated with each other unlike other symptom clusters in the ITQ (Knefel et al., 2019; McElroy et al., 2019). Results also exhibited a particularly low reliability in the affective dysregulation subscale from the ITQ-12 and ITQ-22, raising concerns about the precision and stability of measurements when assessing affect dysregulation as a unitary construct. While affective dysregulation is a pivotal and theoretically intricate challenge in CPTSD (Sele et al., 2020), treating affective hyperactivation and hypoactivation as distinct has potential benefits to enhance construct validity of CPTSD (Kindred et al., 2024) and incorporating these dimensions could lead to more accurate diagnoses. Indeed, recommendations for the ICD-11 working group prior to its completion included that the affective dysregulation is best represented by hyperactivation and hypoactivation dimensions (Ben-Ezra et al., 2018; Shevlin et al., 2018). In addition, there may be benefits in the clinical utility of psychotherapeutic treatment of CPTSD in separating these constructs (Frewen et al., 2023). Parallel to the longstanding discussion regarding phase-based or single-phase interventions for CPTSD, chronological sequencing of intervention strategies may be of clinical importance as different strategies may address these distinct types of dysregulation. For instance, Skills Training in Affective and Interpersonal Regulation (STAIR) Narrative Therapy (Cloitre et al., 2006) is a structured cognitive-behavioral therapy that teaches appropriate emotion regulation skills before trauma memory processing to improve daily and relational functioning and enhance the effectiveness of exposure therapy.

While there was clear superiority of the seven correlated first-order factor model, the two-factor second-order model demonstrated a good fit, and for this reason, it should not be dismissed. Mixture models consistently reveal the existence of distinct classes. All of the 13 cluster analyses reviewed in Redican et al. (2021), indicated a “PTSD” and a “CPTSD” class, which is congruent with the two-factor second-order model which differentiates between core PTSD and DSO factors. However, it is crucial to recognize the limitations of cluster analyses; while they identify statistical groupings rather than underlying latent dimensions, there is consensus that cluster analyses alone cannot confirm the validity of a diagnostic construct (Rød & Schmidt, 2021). A comprehensive understanding of PTSD and CPTSD requires triangulating evidence across diverse methodologies, such as factor analysis, network analyses, cluster analysis, and mixture models, to establish a robust and convergent foundation for their diagnostic validity.

The lack of superiority for the two-factor second-order model could be attributed to both the potential under specification of the ITQ-12 or the ICD-11 diagnostic criteria’s insufficient coverage of complex symptomatology, diminishing clear distinctions between PTSD and CPTSD in factorial analyses. Conducting the analysis on the ITQ-22, which has greater coverage of the ICD-11 diagnostic criteria, might be expected to indicate the superiority of the two-factor second-order model. However, this was not the case and, as such, it may be that the primary limitation of the ITQ lies in the diagnostic criteria it represents. If the criteria are not well-defined or if there are conceptual issues with the way they are formulated, this will impact the results of factor analyses. Some authors posit that symptom profiles of patients with PTSD and CPTSD exhibit a more diverse range than is captured solely by the criteria for core PTSD and DSO (e.g., Ford, 2020). Consequently, the existing measures for ICD-11 PTSD and CPTSD may not yet provide the most precise representation of the constructs they are intended to assess. While some authors (e.g., Frewen et al., 2023) have advocated for more comprehensive assessments to capture the full complexity of trauma responses, we argue that a more refined conceptualization of CPTSD—and improved measurement tools—are needed to accurately reflect the disorder’s structure and ensure reliability. Although the ITQ was designed for clinical utility, its effectiveness will be limited if it fails to reliably measure symptom clusters, leading to inaccurate diagnoses and difficulty distinguishing CPTSD from other disorders, ultimately diminishing its value as a clinical tool. Given that CPTSD is relatively new in its inclusion in official nosology, it is anticipated that, like other mental health diagnoses, it will undergo continued refinement and development. However, despite the pernicious and complex nature of this disorder, the ITQ-12 is a remarkedly strong and brief measure, suitable for assessing ICD-11 PTSD and CPTSD at the factor level.

### Moderator Analysis of Model Fit

The seven correlated first-order factor model was the best-fitting model in PTSD and non-PTSD samples in addition to samples that met probable CPTSD thresholds and those that did not. Redican et al. (2021) found evidence that model fit was moderated by sample type: the first-order model fits best in most community samples, while the second-order model was superior in clinical samples. As qualitative reviews have no good mechanism for assessing the consistency of effects (Borenstein et al., 2009), the discrepancies between these results may be due to the current quantitative meta-analysis providing a more precise estimate of the factorial structure and relationships between factors. However, the limited overlap in shared clinical studies between Redican et al. (2021) and the current quantitative review—which required availability of an inter-item correlation matrix—suggests that other factors such as random sample bias, responder bias, and variations in the types of trauma-exposed populations in these studies, might also influence the results. Briefly, the meta-regression of factor loadings showed statistically significant moderation effects, but their minimal impact on factor loadings suggests the first-order model remains a stable representation of the ITQ structure across samples.

### Reliability of the Best-Fitting Model and the ICD-11 Model

The reliability estimates for several subscales using the seven correlated first-order factor model fall below acceptable levels, potentially compromising the precision of both the ITQ-12 and ITQ-22. This issue is especially relevant for PTSD samples and those with probable CPTSD, as the ITQ-12 tends to be less reliable for these populations. Utilizing a minimal number of symptom indicators, the ITQ-12 was developed to prioritize clinical utility (Shevlin et al., 2018); however, this approach has led to a compromise in composite reliability. This issue is particularly notable in the affective dysregulation factor, which reflects a multifaceted construct where distinct trauma types may correspond to specific patterns of dysregulation. Should the subscale fail to encompass the entire spectrum of these variations, it could impact its reliability. When using the ITQ-12, researchers should be cautioned that using the sum score of the two items under one factor is not a reliable way to assess the factor. Adding or replacing items with more well-designed items for each factor would likely increase the reliability of the sum score. This issue provides a compelling rationale to employ the ITQ-22 instead. While the ITQ-12—which has a total of 18 items combined due to functional impairment items—is suggested to lessen the burden on respondents, it’s doubtful that this reduction is significantly more efficient compared to the 22-item ITQ (Sele et al., 2020). While using the ITQ-22 may somewhat mitigate the lower reliability estimates using a first-order factor model, further revision of items of within its subscales is suggested, as discussed below.

Results indicated good to excellent internal consistency for the two-factor second-order model when using the ITQ-12 and ITQ-22. It is important to note, that this does not undermine the superiority of the seven correlated first-order factor model. However, utilizing the second-order core PTSD and DSO factors may be advantageous, particularly until the conceptualization and criteria of CPTSD are refined, with future measures being more precise in line with these improvements. For instance, implementing the two-factor second-order model could be beneficial with; clinical populations, where the goal is to provide consistent and reliable diagnoses of PTSD and CPTSD; research contexts, facilitating comparisons across different populations or interventions and reducing the risk of measurement error; and, in sample-specific contexts, such as when assessing CPTSD across diverse cultural groups, ensuring that the diagnostic criteria are applicable and interpretable where symptom expression may vary.

### Clinical and Research Implications

The present results suggest that researchers can feel confident in fitting the first-order factor model of both the finalized and preliminary versions of the ITQ. These models are also supported by clinician-administered measures of CPTSD and in populations we did not consider. For instance, International Trauma Interview (ITI), a new clinician-administered diagnostic interview for ICD-11 PTSD and CPTSD (Gelezelyte et al., 2022), found first- and second-order factor models to be of good fit, along with factor analyses of the ITQ using samples of children and adolescents (Haselgruber et al., 2020; Kazlauskas et al., 2020). Owing to concerns regarding internal consistency, researchers intending to utilize subscales (i.e., first-order factor models) for a more detailed examination of CPTSD should contemplate employing the ITQ-22. This may be particularly useful in PTSD populations and among individuals anticipated to meet CPTSD thresholds.

One way to enhance clinical utility may be to revise the phrasing of items on the ITQ to more accurately reflect the characteristics of CPTSD populations. In one study, the consistency between the ITI and the ITQ for multiple subscales, such as sense of threat, hyperactivation and hypoactivation was poor and the ITQ-12 may yield false positives in probable CPTSD diagnoses (Gelezelyte et al., 2022). This inconsistency between the ITQ, a self-report measure, and the ITI, a clinician-rated CPTSD instrument, may stem from various factors. Some researchers attribute this finding to variations in the sensitivity and specificity of the ITQ (Gelezelyte et al., 2022; Gerrmann et al., 2024) and subsequently suggest modifying the diagnostic algorithms or adjusting the threshold scores to improve its accuracy. However, as our findings suggest that the reliability of the ITQ-12 decreases in clinical contexts and among individuals with more severe CPTSD, the need for the rephrasing of items to more accurately reflect higher symptom severity may be more appropriate. Other potential methods for improving clinical utility could include retaining more relevant items, expanding or adding new items that better represent the phenomenology of CPTSD.

In-depth investigation into contributing factors impacting the superiority of the seven correlated first-order factor model is warranted, exploring whether it originates from diagnostic criteria, operationalization of the constructs within the ITQ, or a combination of both. For instance, external validation of the ITQ could be assessed by comparing subscale scores with measures that theoretically capture distinct aspects of CPTSD. Gerrmann et al. (2024) suggest that items in the DSO scale measure concepts may be closely related, implying its reliability might arise from construct overlap. The distinctiveness of the measured concepts could be further explored by extending the examination of discriminant validity through multiple comparison measures beyond the CPTSD symptoms. Content validity could be further analyzed by assessing the content of the ITQ items to ensure they comprehensively cover the constructs as defined in the ICD-11 criteria. This could involve qualitative methods such as interviews with subject matter experts and focus groups with clinicians. Lastly, comparing the effectiveness of the ITQ-12 versus ITQ-22 in different populations to understand how changes in operationalization affect the performance of this measure is warranted.

A critical examination and refinement of diagnostic criteria to enhance the concordance and validity of measurements in line with the conceptualization of CPTSD is warranted. PTSD—of which, symptoms continue to be debated—has been included in the DSM since 1980. However, CPTSD is a relatively recent addition to nosology and, as such, it is to be expected that the CPTSD construct will require further revision. Similarly to how the PTSD Checklist for DSM-5 (Weathers et al., 2018) has been updated with new editions of the DSM; the ITQ could update itself along with ongoing iterations of the ICD, which would hypothetically result in further construct validity.

An important avenue for future research involves examining the relationships between the ITQ factors and functional impairment items of ICD-11 PTSD and CPTSD. While these items were excluded from the present factor analyses due to their non-contribution to the dimensional structure, their associations with the ITQ factors could offer valuable insights into the clinical relevance and utility of these constructs. Incorporating functional impairment enhances the ecological validity of diagnoses by aligning them with the practical realities of how disorders affect daily life.

Future research could investigate the implications of splitting the affective dysregulation factor into affective hyperactivation and affective hypoactivation. From a psychometric standpoint, there is a need for future research to fine-tune the affective dysregulation facet, potentially by incorporating additional items to mitigate the impact of measurement error and better focus on the domain of the construct. Ongoing exploration is warranted to elucidate the nuances and implications of considering affective dysregulation as a unitary or dual construct. This meta-analysis, along with a mounting evidence base, indicates that future iterations of the ICD formulation of CPTSD should include specification of the affective hyperactivation and hypoactivation dimensions, as suggested by a number of CPTSD researchers (Cloitre et al., 2020).

### Limitations

There are several potential limitations in this study. First, the results could also be influenced by the characteristics of the sample used in the analysis. Most of the studies focused on non-PTSD groups or those not meeting the criteria for CPTSD. Nonetheless, given the absence of any substantial factorial variance among these different groups, the disparity in the sample characteristics is unlikely to significantly alter the findings. Second, the inability to incorporate continuous moderators and the lack of support for the simultaneous inclusion of multiple moderating variables are acknowledged constraints of the TSSEM approach. This limitation renders the moderator analyses susceptible to potential confounding factors. Moderator analyses are particularly important as the large range of heterogeneity in correlations and underscore the importance of caution when generalizing finding. The focus of this study was on model fit, specifically examining the factorial structure of the ITQ. In contrast, the moderation of specific parameters, such as factor loadings, assesses whether these parameters vary systematically across levels of a moderator. Future research could explore both moderation of model fit and parameter moderation. Primary data studies and individual participant data meta-analyses allow for the simultaneous testing of multiple moderators and may mitigate biases introduced by study-level aggregation, providing a more accurate and nuanced understanding of heterogeneity in model fit and parameters.

Third, using a random-effects analysis in the PTSD and probable CPTSD subgroups was unattainable due to the sample size. Using a fixed-effects model for these subgroups implies that the results found in our sample of studies may not be generalizable to other or future studies. However, the parameter estimates obtained from the fixed-effects analysis are based on a wide range of studies, as there was a large diversity of sample characteristics across gender, age, cultural background, language used for ITQ administration, trauma type, and geographical locations.

Fourth, despite efforts to obtain unpublished data, our search may not have fully captured gray literature. Although additional studies are unlikely to substantially alter the results, minimizing this risk remains important. Future reviews may benefit from including platforms such as Google Scholar and open data repositories (e.g., OSF).

The focus of this paper was on evaluating the structural properties and reliability of the ITQ within its standardized framework, not on latent factor variances or invariance testing. We acknowledge that analyzing covariance matrices would allow for measurement invariance analyses across groups, addressing differences in factor loadings and latent variances without the potential confounds associated with correlation matrices. This represents a valuable avenue for future research, particularly for exploring the consistency of the ITQ’s factor structures across diverse populations and contexts. Researchers may leverage the covariance matrices collected for this study to test metric invariance or to conduct more nuanced confirmatory analyses that incorporate latent factor variance differences.

## Conclusion

The seven correlated first-order factor model provides a strong empirical explanation for the ITQ item correlation structure, providing robust factor loadings. Other models—particularly the two-factor second-order model aligned with the ICD-11 conceptualization—also demonstrated good fit and remain relevant for consideration. This analysis quantitatively synthesized data from 43,066 individuals across 31 countries testing 9 factorial models. Due to the preciseness and comprehensiveness of this research, which exceeds any previous analyses, numerous research questions and discussions concerning the validity of the ICD-11 PTSD and CPTSD diagnoses have been effectively addressed. The two-item subscales of the ITQ that are used to measure first-order dimensions exhibit limited reliability. Using a subscale with more items to measure higher order factors offers adequate reliability, for example, by using the scores from the final six items to measure DSO. However, enhancing diagnostic precision and ensuring more comprehensive coverage of CPTSD may improve the ability of measures to distinguish between PTSD and CPTSD within hierarchical models. One promising avenue, supported by accumulating evidence, involves the differentiation of affective dysregulation into hyperactivation and hypoactivation.

In summary, this study affirms the adequacy of the ITQ as a tool for assessing ICD-11 PTSD and CPTSD, providing strong support for its factorial validity across diverse samples. However, additional research is necessary to enhance the construct validity ICD PTSD and CPTSD and to account for variation in symptom profiles of trauma-effected individuals that may not be currently reflected by the current diagnostic criteria alone.

## Supplemental Material

sj-docx-1-asm-10.1177_10731911251340837 – Supplemental material for Evaluating the ICD-11 PTSD and Complex PTSD Constructs Supplemental material, sj-docx-1-asm-10.1177_10731911251340837 for Evaluating the ICD-11 PTSD and Complex PTSD Constructs by Reuben Kindred, Suzanne Jak, Ruby Hamer, Maja Nedeljkovic and Glen W. Bates in Assessment
