# Supplementary material for: Evaluating the ICD-11 PTSD and Complex PTSD Constructs: A Meta-Analytic Confirmatory Factor Analysis of the International Trauma Questionnaire
Source: Assessment. 2025 May 30;33(4):510–32. doi: 10.1177/10731911251340837 (PMC13153442; doi:10.1177/10731911251340837)
Supplement: sj-docx-1-asm-10.1177_10731911251340837 – Supplemental material for Evaluating the ICD-11 PTSD and Complex PTSD Constructs [file sj-docx-1-asm-10.1177_10731911251340837.docx]

**Supplementary Material**

**Supplementary Figure 1.** Factor models for the preliminary International Trauma Questionnaire (ITQ-22)


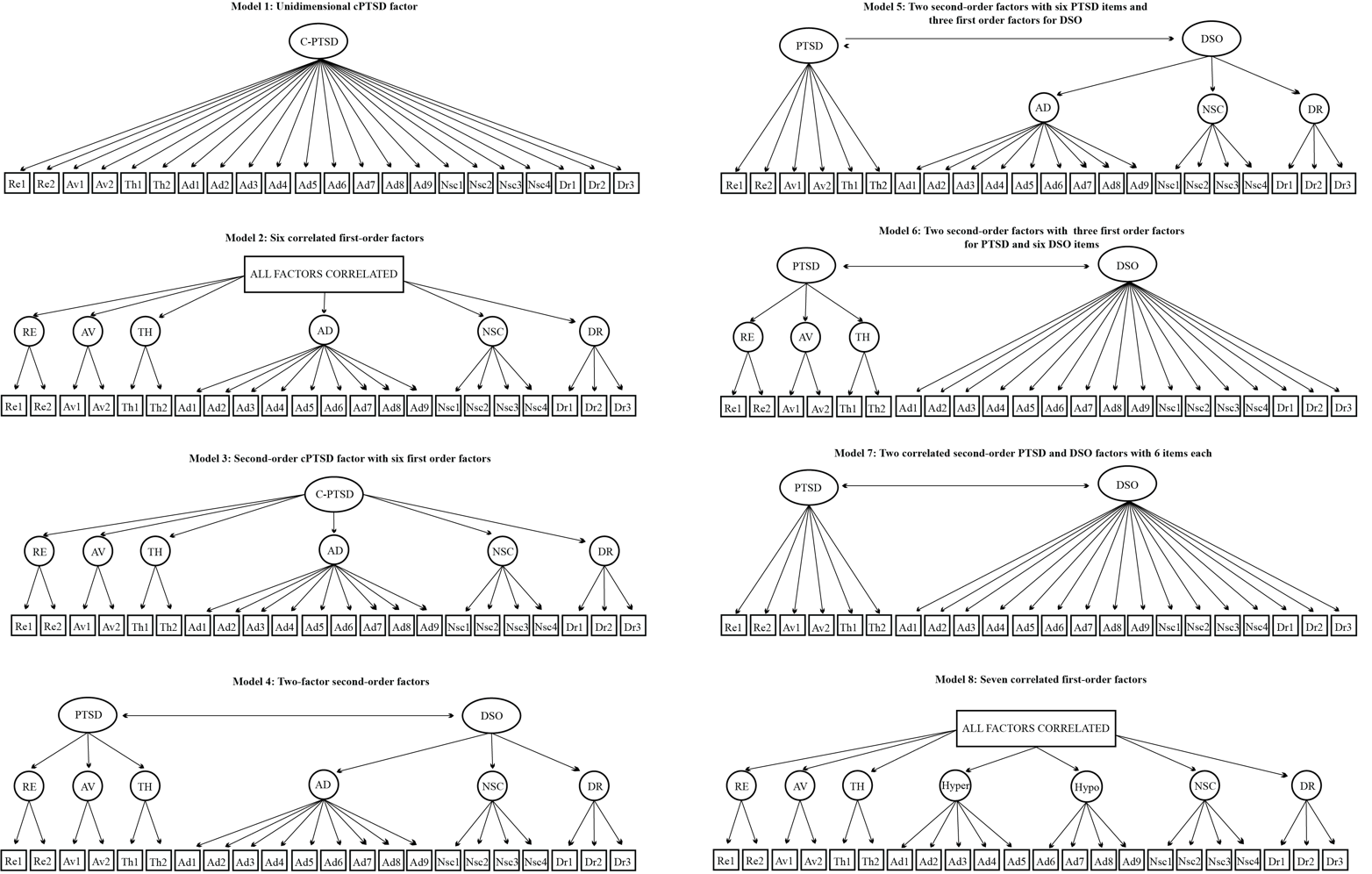


**
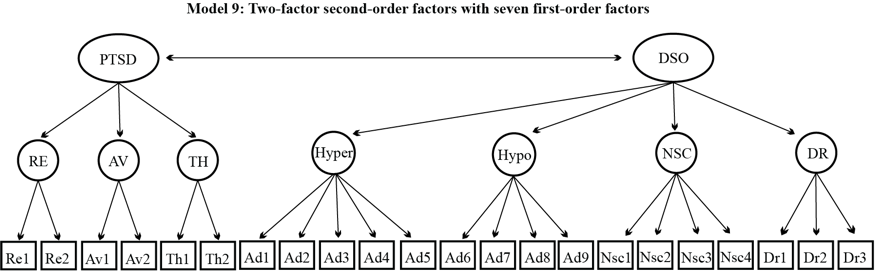
**

**Supplementary Figure 2.** Standardised factor loadings for models reaching good fit for the International Trauma Questionnaire (ITQ-22)


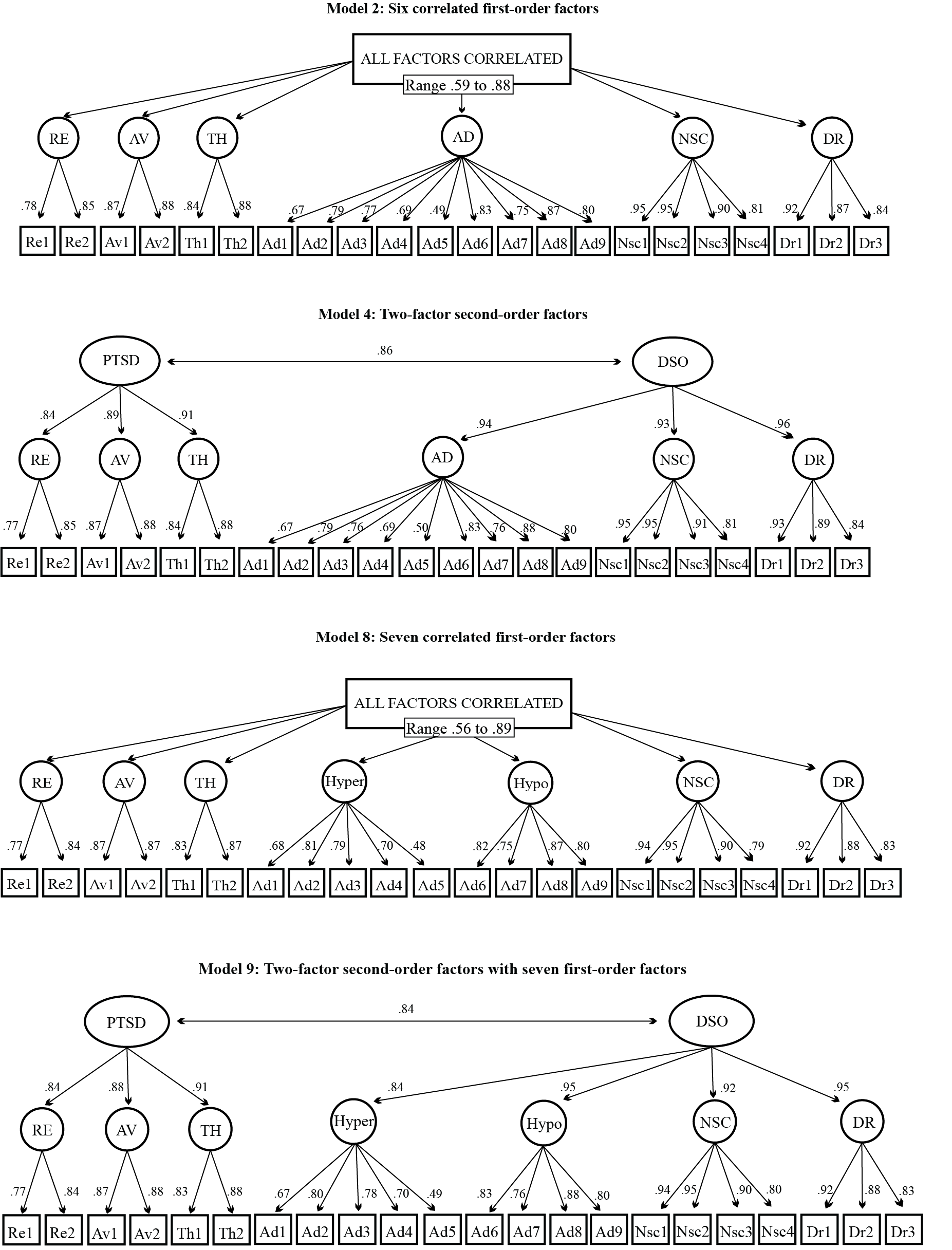


**Supplementary Table 1.** PRISMA Checklist

| **Section and Topic** | **Item #** | **Checklist item** | **Location where item is reported** |
| --- | --- | --- | --- |
| **TITLE** | | |  |
| Title | 1 | Identify the report as a systematic review. | Page 1-2 |
| **ABSTRACT** | | |  |
| Abstract | 2 | See the PRISMA 2020 for Abstracts checklist. | Page 2 |
| **INTRODUCTION** | | |  |
| Rationale | 3 | Describe the rationale for the review in the context of existing knowledge. | Pages 3-5 |
| Objectives | 4 | Provide an explicit statement of the objective(s) or question(s) the review addresses. | Page 5-6 |
| **METHODS** | | |  |
| Eligibility criteria | 5 | Specify the inclusion and exclusion criteria for the review and how studies were grouped for the syntheses. | Page 7 |
| Information sources | 6 | Specify all databases, registers, websites, organisations, reference lists and other sources searched or consulted to identify studies. Specify the date when each source was last searched or consulted. | Page 6 |
| Search strategy | 7 | Present the full search strategies for all databases, registers and websites, including any filters and limits used. | Page 6 and Supplementary Table 2 |
| Selection process | 8 | Specify the methods used to decide whether a study met the inclusion criteria of the review, including how many reviewers screened each record and each report retrieved, whether they worked independently, and if applicable, details of automation tools used in the process. | Pages 6-7 |
| Data collection process | 9 | Specify the methods used to collect data from reports, including how many reviewers collected data from each report, whether they worked independently, any processes for obtaining or confirming data from study investigators, and if applicable, details of automation tools used in the process. | Page 8 |
| Data items | 10a | List and define all outcomes for which data were sought. Specify whether all results that were compatible with each outcome domain in each study were sought (e.g. for all measures, time points, analyses), and if not, the methods used to decide which results to collect. | Page 9 |
|  | 10b | List and define all other variables for which data were sought (e.g. participant and intervention characteristics, funding sources). Describe any assumptions made about any missing or unclear information. | Page 8 |
| Study risk of bias assessment | 11 | Specify the methods used to assess risk of bias in the included studies, including details of the tool(s) used, how many reviewers assessed each study and whether they worked independently, and if applicable, details of automation tools used in the process. | Page 9 |
| Effect measures | 12 | Specify for each outcome the effect measure(s) (e.g. risk ratio, mean difference) used in the synthesis or presentation of results. | Page 10 |
| Synthesis methods | 13a | Describe the processes used to decide which studies were eligible for each synthesis (e.g. tabulating the study intervention characteristics and comparing against the planned groups for each synthesis (item #5)). | Page 8 |
|  | 13b | Describe any methods required to prepare the data for presentation or synthesis, such as handling of missing summary statistics, or data conversions. | Page 11 |
|  | 13c | Describe any methods used to tabulate or visually display results of individual studies and syntheses. | N/A |
|  | 13d | Describe any methods used to synthesize results and provide a rationale for the choice(s). If meta-analysis was performed, describe the model(s), method(s) to identify the presence and extent of statistical heterogeneity, and software package(s) used. | Pages 10-12  Page 14 |
|  | 13e | Describe any methods used to explore possible causes of heterogeneity among study results (e.g. subgroup analysis, meta-regression). | Pages 12-13 |
|  | 13f | Describe any sensitivity analyses conducted to assess robustness of the synthesized results. | Page 12 |
| Reporting bias assessment | 14 | Describe any methods used to assess risk of bias due to missing results in a synthesis (arising from reporting biases). | Page 12 |
| Certainty assessment | 15 | Describe any methods used to assess certainty (or confidence) in the body of evidence for an outcome. | Page 12 |
| **RESULTS** | | |  |
| Study selection | 16a | Describe the results of the search and selection process, from the number of records identified in the search to the number of studies included in the review, ideally using a flow diagram. | Page 7 |
|  | 16b | Cite studies that might appear to meet the inclusion criteria, but which were excluded, and explain why they were excluded. | Page 11 |
| Study characteristics | 17 | Cite each included study and present its characteristics. | Pages 16-19 |
| Risk of bias in studies | 18 | Present assessments of risk of bias for each included study. | Supplementary Table 9 |
| Results of individual studies | 19 | For all outcomes, present, for each study: (a) summary statistics for each group (where appropriate) and (b) an effect estimate and its precision (e.g. confidence/credible interval), ideally using structured tables or plots. | (a) Page 23 |
| Results of syntheses | 20a | For each synthesis, briefly summarise the characteristics and risk of bias among contributing studies. | Pages 23-24 |
|  | 20b | Present results of all statistical syntheses conducted. If meta-analysis was done, present for each the summary estimate and its precision (e.g. confidence/credible interval) and measures of statistical heterogeneity. If comparing groups, describe the direction of the effect. | Pages 20-27  Supplementary Table 14. |
|  | 20c | Present results of all investigations of possible causes of heterogeneity among study results. | Page 23; 27-29 |
|  | 20d | Present results of all sensitivity analyses conducted to assess the robustness of the synthesized results. | Supplementary Tables 11-13 |
| Reporting biases | 21 | Present assessments of risk of bias due to missing results (arising from reporting biases) for each synthesis assessed. | Supplementary Table 12 |
| Certainty of evidence | 22 | Present assessments of certainty (or confidence) in the body of evidence for each outcome assessed. | Page 15 |
| **DISCUSSION** | | |  |
| Discussion | 23a | Provide a general interpretation of the results in the context of other evidence. | Pages 29-34 |
|  | 23b | Discuss any limitations of the evidence included in the review. | Page 37 |
|  | 23c | Discuss any limitations of the review processes used. | Pages 38-39 |
|  | 23d | Discuss implications of the results for practice, policy, and future research. | Pages 35-37 |
| **OTHER INFORMATION** | | |  |
| Registration and protocol | 24a | Provide registration information for the review, including register name and registration number, or state that the review was not registered. | Page 6 |
|  | 24b | Indicate where the review protocol can be accessed, or state that a protocol was not prepared. | Page 6 |
|  | 24c | Describe and explain any amendments to information provided at registration or in the protocol. | Not required |
| Support | 25 | Describe sources of financial or non-financial support for the review, and the role of the funders or sponsors in the review. | Reported at journal submission. |
| Competing interests | 26 | Declare any competing interests of review authors. | Not required |
| Availability of data, code and other materials | 27 | Report which of the following are publicly available and where they can be found: template data collection forms; data extracted from included studies; data used for all analyses; analytic code; any other materials used in the review. | Page 14 |

**Supplementary Table 2.** Search strategy

| **Database** | **Search String** | **Hits** | **Date** |
| --- | --- | --- | --- |
| CINAHL | “CPTSD” OR “C-ptsd” OR “Complex PTSD” OR “Complex Post traumatic stress” OR “complex trauma disorder” OR “disturbances in self-organization,” OR “International Trauma Questionnaire” (Title/Abstract) | 187  44 | 01/04/2023  22/04/2024 |
| Cochrane | “CPTSD” OR “C-ptsd” OR “Complex PTSD” OR “Complex Post traumatic stress” OR “complex trauma disorder” OR “disturbances in self-organization,” OR “International Trauma Questionnaire” (Title or Abstract or Keyword) | 75  24 | 01/04/2023  22/04/2024 |
| MEDLINE | “CPTSD” OR “C-ptsd” OR “Complex PTSD” OR “Complex Post traumatic stress” OR “complex trauma disorder” OR “disturbances in self-organization,” OR “International Trauma Questionnaire” (Topic)  Specifier: Journal Article, Validation study, Research support, Clinical Trial | 455  131 | 01/04/2023  22/04/2024 |
| Proquest Dissertations + Theses | “CPTSD” OR “C-ptsd” OR “Complex PTSD” OR “Complex Post traumatic stress” OR “complex trauma disorder” OR “disturbances in self-organization,” OR “International Trauma Questionnaire” (Title/Abstract) | 83  20 | 01/04/2023  22/04/2024 |
| PsycNET | “CPTSD” OR “C-ptsd” OR “Complex PTSD” OR “Complex Post traumatic stress” OR “complex trauma disorder” OR “disturbances in self-organization,” OR “International Trauma Questionnaire” (Title/Abstract) Specifier: Journal | 472  132 | 01/04/2023  22/04/2024 |
| Pubmed | “CPTSD” OR “C-ptsd” OR “Complex PTSD” OR “Complex Post traumatic stress” OR “complex trauma disorder” OR “disturbances in self-organization,” OR “International Trauma Questionnaire” (Title/Abstract) | 481  150 | 01/04/2023  22/04/2024 |
|  |  |  |  |
|  |  |  |  |
| Scopus | “CPTSD” OR “C-ptsd” OR “Complex PTSD” OR “Complex Post traumatic stress” OR “complex trauma disorder” OR “disturbances in self-organization,” OR “International Trauma Questionnaire” (Document title or Abstract)  Source type: Dissertations and Thesis | 560  235 | 01/04/2023  22/04/2024 |
| Web of Science | “CPTSD” OR “C-ptsd” OR “Complex PTSD” OR “Complex Post traumatic stress” OR “complex trauma disorder” OR “disturbances in self-organization,” OR “International Trauma Questionnaire” | 607  193 | 01/04/2023  22/04/2024 |

**Supplementary Table 3.** Items in each ITQ measure

| **Code** | **Item** | **Measure inclusion** |
| --- | --- | --- |
| **Re1** | Having upsetting dreams that replay part of the experience or are clearly related to the experience | ITQ-12, ITQ-14a, ITQ-14b, ITQ-20, ITQ-22 |
| **Re2** | Having powerful images or memories that sometimes come into your mind in which you feel the experience is happening again in the here and now | ITQ-12, ITQ-14a, ITQ-14b, ITQ-20, ITQ-22 |
| **Av1** | Avoiding internal reminders of the experience (for example, thoughts, feelings or physical sensations) | ITQ-12, ITQ-14a, ITQ-14b, ITQ-20, ITQ-22 |
| **Av2** | Avoiding external reminders of the experience (for example, people, places, conversations, objects, activities or situations) | ITQ-12, ITQ-14a, ITQ-14b, ITQ-20, ITQ-22 |
| **Th1** | Being ‘super-alert’, watchful, or on guard | ITQ-12, ITQ-14a, ITQ-14b, ITQ-20, ITQ-22 |
| **Th2** | Feeling jumpy or easily startled | ITQ-12, ITQ-14a, ITQ-14b, ITQ-20, ITQ-22 |
| **Ad1** | I react intensely to things that do not seem to affect other people so much | ITQ-14a, ITQ-20, ITQ-22 |
| **Ad2** | When I am upset, it takes me a long time to calm down | ITQ-12, ITQ-14a, ITQ-14b, ITQ-20, ITQ-22 |
| **Ad3** | My feelings tend to be easily hurt | ITQ-14b, ITQ-20, ITQ-22 |
| **Ad4** | I experience episodes of uncontrollable anger | ITQ-22 |
| **Ad5** | I do things that people have told me are dangerous or reckless | ITQ-22 |
| **Ad6** | I feel numb or emotionally shut down | ITQ-12, ITQ-14a, ITQ-14b, ITQ-20, ITQ-22 |
| **Ad7** | I am the kind of person who has difficulty experiencing feelings of pleasure or joy | ITQ-14a, ITQ-14b, ITQ-20, ITQ-22 |
| **Ad8** | When I am under stress or confronted with reminders of my trauma, I often feel that the world is distant or that the world seems different | ITQ-20, ITQ-22 |
| **Ad9** | When I am under stress or confronted with reminders of my trauma, I often feel outside my body or feel that there is something strange about my body | ITQ-20, ITQ-22 |
| **Nsc1** | I feel like a failure | ITQ-12, ITQ-14a, ITQ-14b, ITQ-20, ITQ-22 |
| **Nsc2** | I feel worthless | ITQ-12, ITQ-14a, ITQ-14b, ITQ-20, ITQ-22 |
| **Nsc3** | I often feel ashamed of myself whether it makes sense or not | ITQ-20, ITQ-22 |
| **Nsc4** | I feel guilty about things I have done or failed to do | ITQ-20, ITQ-22 |
| **Dr1** | I feel distant or cut-off from people | ITQ-12, ITQ-14a, ITQ-14b, ITQ-20, ITQ-22 |
| **Dr2** | I find it hard to stay emotionally close to people | ITQ-12, ITQ-14a, ITQ-14b, ITQ-20, ITQ-22 |
| **Dr3** | I avoid relationships because they end up being too difficult or painful | ITQ-20, ITQ-22 |
| *Note.* Ad, Affect dysregulation; Av; Avoidance; Dr, Disturbances in relationships; ITQ, International Trauma Questionnaire; Nsc, Negative self-concept; Re, Re-experiencing; Th; Sense of threat. | | |

**Supplementary Table 4.** Number of studies/total sample sizes for each coefficient in the variance/covariance matrix of the ITQ-12

|  | **Re1** | **Re2** | **Av1** | **Av2** | **Th1** | **Th2** | **Ad1** | **Ad2** | **Nsc1** | **Nsc2** | **Dr1** | **Dr2** |
| --- | --- | --- | --- | --- | --- | --- | --- | --- | --- | --- | --- | --- |
| **Re1** | 55/37,921 | 55/37,921 | 55/37,921 | 55/37,921 | 55/37,921 | 55/37,921 | 55/37,921 | 55/37,921 | 55/37,921 | 55/37,921 | 55/37,921 | 55/37,921 |
| **Re2** | 55/37,921 | 55/37,921 | 55/37,921 | 55/37,921 | 55/37,921 | 55/37,921 | 55/37,921 | 55/37,921 | 55/37,921 | 55/37,921 | 55/37,921 | 55/37,921 |
| **Av1** | 55/37,921 | 55/37,921 | 55/37,921 | 55/37,921 | 55/37,921 | 55/37,921 | 55/37,921 | 55/37,921 | 55/37,921 | 55/37,921 | 55/37,921 | 55/37,921 |
| **Av2** | 55/37,921 | 55/37,921 | 55/37,921 | 55/37,921 | 55/37,921 | 55/37,921 | 55/37,921 | 55/37,921 | 55/37,921 | 55/37,921 | 55/37,921 | 55/37,921 |
| **Th1** | 55/37,921 | 55/37,921 | 55/37,921 | 55/37,921 | 55/37,921 | 55/37,921 | 55/37,921 | 55/37,921 | 55/37,921 | 55/37,921 | 55/37,921 | 55/37,921 |
| **Th2** | 55/37,921 | 55/37,921 | 55/37,921 | 55/37,921 | 55/37,921 | 55/37,921 | 55/37,921 | 55/37,921 | 55/37,921 | 55/37,921 | 56/42,768 | 56/42,768 |
| **Ad1** | 55/37,921 | 55/37,921 | 55/37,921 | 55/37,921 | 55/37,921 | 55/37,921 | 58/43,066 | 57/42,954 | 58/43,066 | 58/43,066 | 57/42,954 | 57/42,954 |
| **Ad2** | 55/37,921 | 55/37,921 | 55/37,921 | 55/37,921 | 55/37,921 | 55/37,921 | 57/42,954 | 57/42,954 | 57/42,954 | 57/42,954 | 57/42,954 | 57/42,954 |
| **Nsc1** | 55/37,921 | 55/37,921 | 55/37,921 | 55/37,921 | 55/37,921 | 55/37,921 | 58/43,066 | 57/42,954 | 58/43,066 | 58/43,066 | 57/42,954 | 57/42,954 |
| **Nsc2** | 55/37,921 | 55/37,921 | 55/37,921 | 55/37,921 | 55/37,921 | 55/37,921 | 58/43,066 | 57/42,954 | 58/43,066 | 58/43,066 | 57/42,954 | 57/42,954 |
| **Dr1** | 55/37,921 | 55/37,921 | 55/37,921 | 55/37,921 | 55/37,921 | 56/42,768 | 57/42,954 | 57/42,954 | 57/42,954 | 57/42,954 | 56/42,735 | 57/42,954 |
| **Dr2** | 55/37,921 | 55/37,921 | 55/37,921 | 55/37,921 | 55/37,921 | 56/42,768 | 57/42,954 | 57/42,954 | 57/42,954 | 57/42,954 | 57/42,954 | 57/42,954 |
| *Note.* Ad, Affect dysregulation; Av; Avoidance; Dr, Disturbances in relationships; Nsc, Negative self-concept; Re, Re-experiencing; Th; Sense of threat; | | | | | | | | | | | | |

**Supplementary Table 5.** Number of studies for each coefficient in the variance/covariance matrix of the ITQ-22

|  | **RE1** | **RE2** | **AV1** | **AV2** | **TH1** | **TH2** | **AD1** | **AD2** | **AD3** | **AD4** | **AD5** | **AD6** | **AD7** | **AD8** | **AD9** | **NSC1** | **NSC2** | **NSC3** | **NSC4** | **DR1** | **DR2** | **DR3** |
| --- | --- | --- | --- | --- | --- | --- | --- | --- | --- | --- | --- | --- | --- | --- | --- | --- | --- | --- | --- | --- | --- | --- |
| **RE1** | 5 | 5 | 5 | 5 | 5 | 5 | 5 | 5 | 5 | 5 | 5 | 5 | 5 | 5 | 5 | 5 | 5 | 4 | 4 | 5 | 5 | 4 |
| **RE2** | 5 | 5 | 5 | 5 | 5 | 5 | 5 | 5 | 5 | 5 | 5 | 5 | 5 | 5 | 5 | 5 | 5 | 4 | 4 | 5 | 5 | 4 |
| **AV1** | 5 | 5 | 5 | 5 | 5 | 5 | 5 | 5 | 5 | 5 | 5 | 5 | 5 | 5 | 5 | 5 | 5 | 4 | 4 | 5 | 5 | 4 |
| **AV2** | 5 | 5 | 5 | 5 | 5 | 5 | 5 | 5 | 5 | 5 | 5 | 5 | 5 | 5 | 5 | 5 | 5 | 4 | 4 | 5 | 5 | 4 |
| **TH1** | 5 | 5 | 5 | 5 | 5 | 5 | 5 | 5 | 5 | 5 | 5 | 5 | 5 | 5 | 5 | 5 | 5 | 4 | 4 | 5 | 5 | 4 |
| **TH2** | 5 | 5 | 5 | 5 | 5 | 5 | 5 | 5 | 5 | 5 | 5 | 5 | 5 | 5 | 5 | 5 | 5 | 4 | 4 | 5 | 5 | 4 |
| **AD1** | 5 | 5 | 5 | 5 | 5 | 5 | 8 | 8 | 8 | 7 | 8 | 7 | 7 | 8 | 7 | 8 | 8 | 6 | 6 | 7 | 7 | 7 |
| **AD2** | 5 | 5 | 5 | 5 | 5 | 5 | 8 | 8 | 8 | 7 | 8 | 7 | 7 | 8 | 7 | 8 | 8 | 6 | 6 | 7 | 7 | 7 |
| **AD3** | 5 | 5 | 5 | 5 | 5 | 5 | 8 | 8 | 8 | 7 | 8 | 7 | 7 | 8 | 7 | 8 | 8 | 6 | 6 | 7 | 7 | 7 |
| **AD4** | 5 | 5 | 5 | 5 | 5 | 5 | 7 | 7 | 7 | 7 | 7 | 7 | 7 | 7 | 7 | 7 | 7 | 6 | 6 | 7 | 7 | 6 |
| **AD5** | 5 | 5 | 5 | 5 | 5 | 5 | 8 | 8 | 8 | 7 | 8 | 7 | 7 | 8 | 7 | 8 | 8 | 6 | 6 | 7 | 7 | 7 |
| **AD6** | 5 | 5 | 5 | 5 | 5 | 5 | 7 | 7 | 7 | 7 | 7 | 7 | 7 | 7 | 7 | 7 | 7 | 6 | 6 | 7 | 7 | 6 |
| **AD7** | 5 | 5 | 5 | 5 | 5 | 5 | 7 | 7 | 7 | 7 | 7 | 7 | 7 | 7 | 7 | 7 | 7 | 6 | 6 | 7 | 7 | 6 |
| **AD8** | 5 | 5 | 5 | 5 | 5 | 5 | 8 | 8 | 8 | 7 | 8 | 7 | 7 | 8 | 7 | 8 | 8 | 6 | 6 | 7 | 7 | 7 |
| **AD9** | 5 | 5 | 5 | 5 | 5 | 5 | 7 | 7 | 7 | 7 | 7 | 7 | 7 | 7 | 7 | 7 | 7 | 6 | 6 | 7 | 7 | 6 |
| **NSC1** | 5 | 5 | 5 | 5 | 5 | 5 | 8 | 8 | 8 | 7 | 8 | 7 | 7 | 8 | 7 | 8 | 8 | 6 | 6 | 7 | 7 | 7 |
| **NSC2** | 5 | 5 | 5 | 5 | 5 | 5 | 8 | 8 | 8 | 7 | 8 | 7 | 7 | 8 | 7 | 8 | 8 | 6 | 6 | 7 | 7 | 7 |
| **NSC3** | 4 | 4 | 4 | 4 | 4 | 4 | 6 | 6 | 6 | 6 | 6 | 6 | 6 | 6 | 6 | 6 | 6 | 6 | 6 | 6 | 6 | 6 |
| **NSC4** | 4 | 4 | 4 | 4 | 4 | 4 | 6 | 6 | 6 | 6 | 6 | 6 | 6 | 6 | 6 | 6 | 6 | 6 | 6 | 6 | 6 | 6 |
| **DR1** | 5 | 5 | 5 | 5 | 5 | 5 | 7 | 7 | 7 | 7 | 7 | 7 | 7 | 7 | 7 | 7 | 7 | 6 | 6 | 6 | 7 | 6 |
| **DR2** | 5 | 5 | 5 | 5 | 5 | 5 | 7 | 7 | 7 | 7 | 7 | 7 | 7 | 7 | 7 | 7 | 7 | 6 | 6 | 7 | 7 | 6 |
| **DR3** | 4 | 4 | 4 | 4 | 4 | 4 | 7 | 7 | 7 | 6 | 7 | 6 | 6 | 7 | 6 | 7 | 7 | 6 | 6 | 6 | 6 | 7 |
| *Note.* Ad, Affect dysregulation; Av; Avoidance; Dr, Disturbances in relationships; Nsc, Negative self-concept; Re, Re-experiencing; Th; Sense of threat. | | | | | | | | | | | | | | | | | | | | | | |

**Supplementary Table 6.** Total sample sizes for each coefficient in the variance/covariance matrix of the ITQ-22

|  | **RE1** | **RE2** | **AV1** | **AV2** | **TH1** | **TH2** | **AD1** | **AD2** | **AD3** | **AD4** | **AD5** | **AD6** | **AD7** | **AD8** | **AD9** | **NSC1** | **NSC2** | **NSC3** | **NSC4** | **DR1** | **DR2** | **DR3** |
| --- | --- | --- | --- | --- | --- | --- | --- | --- | --- | --- | --- | --- | --- | --- | --- | --- | --- | --- | --- | --- | --- | --- |
| **RE1** |  | 2601 | 2601 | 2601 | 2601 | 2601 | 2601 | 2601 | 2601 | 2601 | 2601 | 2601 | 2601 | 2601 | 2601 | 2601 | 2601 | 2288 | 2288 | 2601 | 2601 | 2288 |
| **RE2** | 2601 | 2601 | 2601 | 2601 | 2601 | 2601 | 2601 | 2601 | 2601 | 2601 | 2601 | 2601 | 2601 | 2601 | 2601 | 2601 | 2601 | 2288 | 2288 | 2601 | 2601 | 2288 |
| **AV1** | 2601 | 2601 | 2601 | 2601 | 2601 | 2601 | 2601 | 2601 | 2601 | 2601 | 2601 | 2601 | 2601 | 2601 | 2601 | 2601 | 2601 | 2288 | 2288 | 2601 | 2601 | 2288 |
| **AV2** | 2601 | 2601 | 2601 | 2601 | 2601 | 2601 | 2601 | 2601 | 2601 | 2601 | 2601 | 2601 | 2601 | 2601 | 2601 | 2601 | 2601 | 2288 | 2288 | 2601 | 2601 | 2288 |
| **TH1** | 2601 | 2601 | 2601 | 2601 | 2601 | 2601 | 2601 | 2601 | 2601 | 2601 | 2601 | 2601 | 2601 | 2601 | 2601 | 2601 | 2601 | 2288 | 2288 | 2601 | 2601 | 2288 |
| **TH2** | 2601 | 2601 | 2601 | 2601 | 2601 | 2601 | 2601 | 2601 | 2601 | 2601 | 2601 | 2601 | 2601 | 2601 | 2601 | 2601 | 2601 | 2288 | 2288 | 2601 | 2601 | 2288 |
| **AD1** | 2601 | 2601 | 2601 | 2601 | 2601 | 2601 | 7761 | 7761 | 7761 | 7649 | 7761 | 7649 | 7649 | 7761 | 7649 | 7761 | 7761 | 7336 | 7336 | 7649 | 7649 | 7448 |
| **AD2** | 2601 | 2601 | 2601 | 2601 | 2601 | 2601 | 7761 | 7761 | 7761 | 7649 | 7761 | 7649 | 7649 | 7761 | 7649 | 7761 | 7761 | 7336 | 7336 | 7649 | 7649 | 7448 |
| **AD3** | 2601 | 2601 | 2601 | 2601 | 2601 | 2601 | 7761 | 7761 | 7761 | 7649 | 7761 | 7649 | 7649 | 7761 | 7649 | 7761 | 7761 | 7336 | 7336 | 7649 | 7649 | 7448 |
| **AD4** | 2601 | 2601 | 2601 | 2601 | 2601 | 2601 | 7649 | 7649 | 7649 | 7649 | 7649 | 7649 | 7649 | 7649 | 7649 | 7649 | 7649 | 7336 | 7336 | 7649 | 7649 | 7336 |
| **AD5** | 2601 | 2601 | 2601 | 2601 | 2601 | 2601 | 7761 | 7761 | 7761 | 7649 | 7761 | 7649 | 7649 | 7761 | 7649 | 7761 | 7761 | 7336 | 7336 | 7649 | 7649 | 7448 |
| **AD6** | 2601 | 2601 | 2601 | 2601 | 2601 | 2601 | 7649 | 7649 | 7649 | 7649 | 7649 | 7649 | 7649 | 7649 | 7649 | 7649 | 7649 | 7336 | 7336 | 7649 | 7649 | 7336 |
| **AD7** | 2601 | 2601 | 2601 | 2601 | 2601 | 2601 | 7649 | 7649 | 7649 | 7649 | 7649 | 7649 | 7649 | 7649 | 7649 | 7649 | 7649 | 7336 | 7336 | 7649 | 7649 | 7336 |
| **AD8** | 2601 | 2601 | 2601 | 2601 | 2601 | 2601 | 7761 | 7761 | 7761 | 7649 | 7761 | 7649 | 7649 | 7761 | 7649 | 7761 | 7761 | 7336 | 7336 | 7649 | 7649 | 7448 |
| **AD9** | 2601 | 2601 | 2601 | 2601 | 2601 | 2601 | 7649 | 7649 | 7649 | 7649 | 7649 | 7649 | 7649 | 7649 | 7649 | 7649 | 7649 | 7336 | 7336 | 7649 | 7649 | 7336 |
| **NSC1** | 2601 | 2601 | 2601 | 2601 | 2601 | 2601 | 7761 | 7761 | 7761 | 7649 | 7761 | 7649 | 7649 | 7761 | 7649 | 7761 | 7761 | 7336 | 7336 | 7649 | 7649 | 7448 |
| **NSC2** | 2601 | 2601 | 2601 | 2601 | 2601 | 2601 | 7761 | 7761 | 7761 | 7649 | 7761 | 7649 | 7649 | 7761 | 7649 | 7761 | 7761 | 7336 | 7336 | 7649 | 7649 | 7448 |
| **NSC3** | 2288 | 2288 | 2288 | 2288 | 2288 | 2288 | 7336 | 7336 | 7336 | 7336 | 7336 | 7336 | 7336 | 7336 | 7336 | 7336 | 7336 | 7336 | 7336 | 7336 | 7336 | 7336 |
| **NSC4** | 2288 | 2288 | 2288 | 2288 | 2288 | 2288 | 7336 | 7336 | 7336 | 7336 | 7336 | 7336 | 7336 | 7336 | 7336 | 7336 | 7336 | 7336 | 7336 | 7336 | 7336 | 7336 |
| **DR1** | 2601 | 2601 | 2601 | 2601 | 2601 | 2601 | 7649 | 7649 | 7649 | 7649 | 7649 | 7649 | 7649 | 7649 | 7649 | 7649 | 7649 | 7336 | 7336 | 7430 | 7649 | 7336 |
| **DR2** | 2601 | 2601 | 2601 | 2601 | 2601 | 2601 | 7649 | 7649 | 7649 | 7649 | 7649 | 7649 | 7649 | 7649 | 7649 | 7649 | 7649 | 7336 | 7336 | 7649 | 7649 | 7336 |
| **DR3** | 2288 | 2288 | 2288 | 2288 | 2288 | 2288 | 7448 | 7448 | 7448 | 7336 | 7448 | 7336 | 7336 | 7448 | 7336 | 7448 | 7448 | 7336 | 7336 | 7336 | 7336 | 7448 |
| *Note.* Ad, Affect dysregulation; Av; Avoidance; Dr, Disturbances in relationships; Nsc, Negative self-concept; Re, Re-experiencing; Th; Sense of threat; | | | | | | | | | | | | | | | | | | | | | | |

**Supplementary Table 7.** Item means of the total sample and each sub-samples for the ITQ-12 analysis

|  | **Re1** | **Re2** | **Av1** | **Av2** | **Th1** | **Th2** | **Ad1** | **Ad2** | **Nsc1** | **Nsc2** | **Dr1** | **Dr2** |
| --- | --- | --- | --- | --- | --- | --- | --- | --- | --- | --- | --- | --- |
| **Total sample** | 1.397 | 1.561 | 1.741 | 1.716 | 1.882 | 1.685 | 1.979 | 1.708 | 1.623 | 1.517 | 1.804 | 1.707 |
| **Clinical** | 2.445 | 2.513 | 2.884 | 2.954 | 2.996 | 2.879 | 2.504 | 2.466 | 2.492 | 2.458 | 2.576 | 2.565 |
| **Non-clinical** | 1.266 | 1.442 | 1.598 | 1.562 | 1.743 | 1.536 | 1.882 | 1.584 | 1.463 | 1.344 | 1.678 | 1.584 |
| **Probable CPTSD** | 2.285 | 2.491 | 2.758 | 2.778 | 2.884 | 2.766 | 2.698 | 2.506 | 2.622 | 2.544 | 2.687 | 2.569 |
| **Not Probable CPTSD** | 1.055 | 1.204 | 1.350 | 1.308 | 1.497 | 1.270 | 1.681 | 1.369 | 1.208 | 1.091 | 1.428 | 1.362 |

**Supplementary Table 8.** Item means of the total sample for the ITQ-22 analysis

|  | **Re1** | **Re2** | **Av1** | **Av2** | **Th1** | **Th2** | **Ad1** | **Ad2** | **Ad3** | **Ad4** | **Ad5** | **Ad6** | **Ad7** | **Ad8** | **Ad9** | **Nsc1** | **Nsc2** | **Nsc3** | **Nsc4** | **Dr1** | **Dr2** | **Dr3** |
| --- | --- | --- | --- | --- | --- | --- | --- | --- | --- | --- | --- | --- | --- | --- | --- | --- | --- | --- | --- | --- | --- | --- |
| **Total sample** | 1.470 | 1.673 | 1.793 | 1.784 | 1.870 | 1.879 | 1.959 | 2.025 | 2.179 | 1.365 | 0.890 | 1.790 | 1.769 | 1.739 | 1.474 | 1.666 | 1.581 | 1.733 | 1.936 | 2.051 | 1.816 | 1.639 |

**Supplementary Table 9.** Quality Assessment for Included Studies

**Supplementary Table 10.**

The pooled correlation matrix of the ITQ-12 (below diagonal) and Higgins *I*^2^ (above the diagonal) from the random effects analysis

|  | **Re1** | **Re2** | **Av1** | **Av2** | **Th1** | **Th2** | **Ad1** | **Ad2** | **Nsc1** | **Nsc2** | **Dr1** | **Dr2** |
| --- | --- | --- | --- | --- | --- | --- | --- | --- | --- | --- | --- | --- |
| **Re1** | - | 0.94 | 0.87 | 0.77 | 0.73 | 0.66 | 0.59 | 0.46 | 0.26 | 0.28 | 0.36 | 0.26 |
| **Re2** | 0.61 | - | 0.90 | 0.84 | 0.76 | 0.75 | 0.44 | 0.56 | 0.21 | 0.11 | 0.15 | 0.19 |
| **Av1** | 0.46 | 0.51 | - | 0.94 | 0.77 | 0.69 | 0.55 | 0.47 | 0.13 | 0.31 | 0.11 | 0.16 |
| **Av2** | 0.45 | 0.49 | 0.69 | - | 0.80 | 0.71 | 0.54 | 0.31 | 0.51 | 0.37 | 0.27 | 0.14 |
| **Th1** | 0.42 | 0.46 | 0.46 | 0.49 | - | 0.94 | 0.65 | 0.40 | 0.49 | 0.45 | 0.40 | 0.38 |
| **Th2** | 0.46 | 0.48 | 0.47 | 0.48 | 0.65 | - | 0.70 | 0.53 | 0.45 | 0.41 | 0.48 | 0.15 |
| **Ad1** | 0.30 | 0.32 | 0.32 | 0.34 | 0.33 | 0.39 | - | 0.84 | 0.50 | 0.50 | 0.66 | 0.77 |
| **Ad2** | 0.31 | 0.33 | 0.36 | 0.35 | 0.33 | 0.37 | 0.42 | - | 0.78 | 0.74 | 0.87 | 0.79 |
| **Nsc1** | 0.28 | 0.30 | 0.33 | 0.33 | 0.30 | 0.37 | 0.42 | 0.52 | - | 0.97 | 0.79 | 0.78 |
| **Nsc2** | 0.28 | 0.30 | 0.33 | 0.32 | 0.29 | 0.37 | 0.41 | 0.51 | 0.84 | - | 0.78 | 0.74 |
| **Dr1** | 0.29 | 0.31 | 0.35 | 0.36 | 0.35 | 0.39 | 0.43 | 0.59 | 0.61 | 0.62 | - | 0.89 |
| **Dr2** | 0.26 | 0.28 | 0.34 | 0.34 | 0.32 | 0.35 | 0.39 | 0.56 | 0.51 | 0.52 | 0.70 | - |
| *Note.* Ad, Affect dysregulation; Av; Avoidance; Dr, Disturbances in relationships; Nsc, Negative self-concept; Re, Re-experiencing; Th; Sense of threat; | | | | | | | | | | | | |

**Supplementary Table 11**. ITQ-12 Model 2 Factor Correlations

|  | Re | Av | Th | Ad | Nsc | Dr |
| --- | --- | --- | --- | --- | --- | --- |
| Re | 1 |  |  |  |  |  |
| Av | 0.73 | 1 |  |  |  |  |
| Th | 0.72 | 0.70 | 1 |  |  |  |
| Ad | 0.61 | 0.62 | 0.65 | 1 |  |  |
| Nsc | 0.44 | 0.50 | 0.52 | 0.78 | 1 |  |
| Dr | 0.41 | 0.44 | 0.46 | 0.75 | 0.90 | 1 |
| *Note:* Ad, Affect dysregulation; Av; Avoidance; Dr, Disturbances in relationships; Nsc, Negative self-concept; Re, Re-experiencing; Th; Sense of threat. | | | | | | |

**Supplementary Table 12.** ITQ-12 Model 8 Factor Correlations

|  | Re | Av | Th | Hyper | Hypo | Nsc | Dr | |
| --- | --- | --- | --- | --- | --- | --- | --- | --- |
| Re | 1 |  |  |  |  |  |  |  |
| Av | 0.75 | 1 |  |  |  |  |  |  |
| Th | 0.72 | 0.72 | 1 |  |  |  |  |  |
| Hyper | 0.46 | 0.46 | 0.54 | 1 |  |  |  |  |
| Hypo | 0.42 | 0.46 | 0.47 | 0.63 | 1 |  |  |  |
| Nsc | 0.46 | 0.52 | 0.54 | 0.71 | 0.76 | 1 |  |  |
| Dr | 0.46 | 0.52 | 0.54 | 0.71 | 0.76 | 0.77 | 1 |  |
| *Note:* Av; Avoidance; Dr, Disturbances in relationships; Hyper, Hyperactivation; Hypo, Hypoactivation; Nsc, Negative self-concept; Re, Re-experiencing; Th; Sense of threat. | | | | | | | |  |

**Supplementary Table 13.** Sensitivity analysis – studies with lack of confirmed back-translation excluded

| **Model** | **χ2** | **DF** | **RMSEA** | **SRMR** | **TLI** | **CFI** | **AIC** | **BIC** |
| --- | --- | --- | --- | --- | --- | --- | --- | --- |
| *Sample: k = 55; N = 37,418* | | | | | | | | |
| Model 1 | 28299.54 | 54 | 0.118 | 0.288 | 0.933 | 0.945 | 28191.54 | 27730.93 |
| Model 2 | 2066.78 | 39 | 0.037 | 0.027 | 0.993 | 0.996 | 1988.78 | 1656.11 |
| Model 3 | 9028.56 | 48 | 0.071 | 0.130 | 0.976 | 0.983 | 8932.56 | 8523.13 |
| Model 4 | 3144.70 | 47 | 0.042 | 0.042 | 0.992 | 0.994 | 3050.70 | 2649.79 |
| Model 5 | 11134.07 | 50 | 0.077 | 0.109 | 0.972 | 0.979 | 11034.07 | 10607.57 |
| Model 6 | 10535.52 | 50 | 0.075 | 0.105 | 0.973 | 0.980 | 10435.52 | 10009.03 |
| Model 7 | 18210.23 | 53 | 0.096 | 0.161 | 0.956 | 0.965 | 18104.23 | 17652.15 |
| **Model 8** | **1272.66** | **33** | **0.032** | **0.017** | **0.995** | **0.998** | **1206.66** | **925.17** |
| Model 9 | 3144.77 | 46 | 0.042 | 0.042 | 0.991 | 0.994 | 3052.77 | 2660.39 |

**Supplementary Table 14.** Sensitivity analysis – studies with low and moderate overall study quality excluded

| **Model** | **χ2** | **DF** | **RMSEA** | **SRMR** | **TLI** | **CFI** | **AIC** | **BIC** |
| --- | --- | --- | --- | --- | --- | --- | --- | --- |
| *Sample: k = 45; N =* 36,676 | | | | | | | | |
| Model 1 | 25159.46 | 54 | 0.113 | 0.291 | 0.945 | 0.955 | 25051.46 | 24591.92 |
| Model 2 | 1727.21 | 39 | 0.034 | 0.026 | 0.995 | 0.997 | 1649.21 | 1317.33 |
| Model 3 | 8023.97 | 48 | 0.067 | 0.137 | 0.980 | 0.986 | 7927.97 | 7519.49 |
| Model 4 | 2662.17 | 47 | 0.039 | 0.040 | 0.994 | 0.995 | 2568.17 | 2168.20 |
| Model 5 | 9744.06 | 50 | 0.073 | 0.109 | 0.977 | 0.983 | 9644.06 | 9218.56 |
| Model 6 | 9636.34 | 50 | 0.072 | 0.099 | 0.977 | 0.983 | 9536.34 | 9110.85 |
| Model 7 | 16439.74 | 53 | 0.092 | 0.156 | 0.964 | 0.971 | 16333.74 | 15882.71 |
| **Model 8** | **1129.92** | **33** | **0.030** | **0.017** | **0.996** | **0.998** | **1063.92** | **783.10** |
| Model 9 | 2664.29 | 46 | 0.039 | 0.040 | 0.993 | 0.995 | 2572.29 | 2180.84 |

**Supplementary Table 15.** Sensitivity analysis – studies with low missingness quality excluded

| **Model** | **χ2** | **DF** | **RMSEA** | **SRMR** | **TLI** | **CFI** | **AIC** | **BIC** |
| --- | --- | --- | --- | --- | --- | --- | --- | --- |
| *Sample: k = 32; N =* 24,073 | | | | | | | | |
| Model 1 | 18072.33 | 54 | 0.118 | 0.285 | 0.935 | 0.947 | 17964.33 | 17527.54 |
| Model 2 | 1306.21 | 39 | 0.037 | 0.027 | 0.994 | 0.996 | 1228.21 | 912.75 |
| Model 3 | 5535.71 | 48 | 0.069 | 0.123 | 0.978 | 0.984 | 5439.71 | 5051.45 |
| Model 4 | 1976.72 | 47 | 0.041 | 0.041 | 0.992 | 0.994 | 1882.72 | 1502.55 |
| Model 5 | 7359.08 | 50 | 0.078 | 0.112 | 0.972 | 0.978 | 7259.08 | 6854.64 |
| Model 6 | 6674.44 | 50 | 0.074 | 0.104 | 0.974 | 0.980 | 6574.44 | 6170.00 |
| Model 7 | 11811.99 | 53 | 0.096 | 0.163 | 0.957 | 0.965 | 11705.99 | 11277.28 |
| **Model 8** | **783.83** | **33** | **0.031** | **0.016** | **0.996** | **0.998** | **717.83** | **450.90** |
| Model 9 | 1977.44 | 46 | 0.042 | 0.041 | 0.992 | 0.994 | 1885.44 | 1513.35 |

|  |  |  |  |  |  |  |
| --- | --- | --- | --- | --- | --- | --- |
|  |  |  |  |  |  |  |
|  |  |  |  |  |  |  |
|  |  |  |  |  |  |  |
|  |  |  |  |  |  |  |
|  |  |  |  |  |  |  |
|  |  |  |  |  |  |  |
|  |  |  |  |  |  |  |
|  |  |  |  |  |  |  |
|  |  |  |  |  |  |  |
|  |  |  |  |  |  |  |
|  |  |  |  |  |  |  |
|  |  |  |  |  |  |  |
|  |  |  |  |  |  |  |
|  |  |  |  |  |  |  |
|  |  |  |  |  |  |  |
|  |  |  |  |  |  |  |
|  |  |  |  |  |  |  |
|  |  |  |  |  |  |  |
|  |  |  |  |  |  |  |

**Supplementary Table 16.** The covariance matrix of the ITQ-22

|  | **RE1** | **RE2** | **AV1** | **AV2** | **TH1** | **TH2** | **AD1** | **AD2** | **AD3** | **AD4** | **AD5** | **AD6** | **AD7** | **AD8** | **AD9** | **NSC1** | **NSC2** | **NSC3** | **NSC4** | **DR1** | **DR2** | **DR3** |
| --- | --- | --- | --- | --- | --- | --- | --- | --- | --- | --- | --- | --- | --- | --- | --- | --- | --- | --- | --- | --- | --- | --- |
| **RE1** | 1 |  |  |  |  |  |  |  |  |  |  |  |  |  |  |  |  |  |  |  |  |  |
| **RE2** | 0.620 | 1 |  |  |  |  |  |  |  |  |  |  |  |  |  |  |  |  |  |  |  |  |
| **AV1** | 0.445 | 0.492 | 1 |  |  |  |  |  |  |  |  |  |  |  |  |  |  |  |  |  |  |  |
| **AV2** | 0.453 | 0.518 | 0.724 | 1 |  |  |  |  |  |  |  |  |  |  |  |  |  |  |  |  |  |  |
| **TH1** | 0.451 | 0.482 | 0.516 | 0.538 | 1 |  |  |  |  |  |  |  |  |  |  |  |  |  |  |  |  |  |
| **TH2** | 0.459 | 0.485 | 0.496 | 0.522 | 0.679 | 1 |  |  |  |  |  |  |  |  |  |  |  |  |  |  |  |  |
| **AD1** | 0.233 | 0.278 | 0.302 | 0.315 | 0.363 | 0.411 | 1 |  |  |  |  |  |  |  |  |  |  |  |  |  |  |  |
| **AD2** | 0.290 | 0.319 | 0.359 | 0.369 | 0.359 | 0.419 | 0.525 | 1 |  |  |  |  |  |  |  |  |  |  |  |  |  |  |
| **AD3** | 0.223 | 0.263 | 0.306 | 0.311 | 0.323 | 0.387 | 0.487 | 0.597 | 1 |  |  |  |  |  |  |  |  |  |  |  |  |  |
| **AD4** | 0.249 | 0.279 | 0.299 | 0.300 | 0.296 | 0.345 | 0.368 | 0.483 | 0.452 | 1 |  |  |  |  |  |  |  |  |  |  |  |  |
| **AD5** | 0.188 | 0.233 | 0.247 | 0.220 | 0.223 | 0.196 | 0.212 | 0.214 | 0.162 | 0.321 | 1 |  |  |  |  |  |  |  |  |  |  |  |
| **AD6** | 0.308 | 0.321 | 0.402 | 0.369 | 0.356 | 0.362 | 0.315 | 0.376 | 0.351 | 0.377 | 0.359 | 1 |  |  |  |  |  |  |  |  |  |  |
| **AD7** | 0.234 | 0.245 | 0.323 | 0.301 | 0.287 | 0.296 | 0.270 | 0.329 | 0.297 | 0.319 | 0.267 | 0.571 | 1 |  |  |  |  |  |  |  |  |  |
| **AD8** | 0.375 | 0.418 | 0.445 | 0.427 | 0.426 | 0.472 | 0.385 | 0.462 | 0.430 | 0.382 | 0.298 | 0.556 | 0.465 | 1 |  |  |  |  |  |  |  |  |
| **AD9** | 0.343 | 0.394 | 0.412 | 0.399 | 0.370 | 0.426 | 0.310 | 0.380 | 0.335 | 0.346 | 0.297 | 0.498 | 0.398 | 0.673 | 1 |  |  |  |  |  |  |  |
| **NSC1** | 0.291 | 0.329 | 0.387 | 0.364 | 0.323 | 0.376 | 0.334 | 0.397 | 0.419 | 0.371 | 0.288 | 0.560 | 0.489 | 0.534 | 0.495 | 1 |  |  |  |  |  |  |
| **NSC2** | 0.294 | 0.331 | 0.399 | 0.378 | 0.324 | 0.404 | 0.331 | 0.396 | 0.418 | 0.360 | 0.285 | 0.550 | 0.488 | 0.532 | 0.488 | 0.871 | 1 |  |  |  |  |  |
| **NSC3** | 0.300 | 0.335 | 0.417 | 0.388 | 0.338 | 0.404 | 0.331 | 0.396 | 0.422 | 0.357 | 0.283 | 0.509 | 0.441 | 0.517 | 0.469 | 0.766 | 0.791 | 1 |  |  |  |  |
| **NSC4** | 0.271 | 0.318 | 0.360 | 0.331 | 0.332 | 0.351 | 0.337 | 0.382 | 0.414 | 0.322 | 0.264 | 0.437 | 0.367 | 0.488 | 0.389 | 0.610 | 0.604 | 0.650 | 1 |  |  |  |
| **DR1** | 0.269 | 0.310 | 0.384 | 0.373 | 0.347 | 0.387 | 0.339 | 0.394 | 0.396 | 0.372 | 0.295 | 0.591 | 0.529 | 0.579 | 0.486 | 0.673 | 0.684 | 0.671 | 0.603 | 1 |  |  |
| **DR2** | 0.256 | 0.284 | 0.374 | 0.360 | 0.336 | 0.336 | 0.279 | 0.346 | 0.353 | 0.342 | 0.276 | 0.561 | 0.571 | 0.485 | 0.413 | 0.564 | 0.572 | 0.560 | 0.497 | 0.723 | 1 |  |
| **DR3** | 0.281 | 0.301 | 0.369 | 0.383 | 0.331 | 0.356 | 0.282 | 0.351 | 0.340 | 0.316 | 0.259 | 0.514 | 0.504 | 0.483 | 0.409 | 0.542 | 0.555 | 0.537 | 0.474 | 0.649 | 0.705 | 1 |
| *Note.* Ad, Affect dysregulation; Av; Avoidance; Dr, Disturbances in relationships; Nsc, Negative self-concept; Re, Re-experiencing; Th; Sense of threat; | | | | | | | | | | | | | | | | | | | | | | |

**Supplementary Table 17.** Model fit statistics for the ITQ-22

| **Model** | **χ2** | **DF** | **RMSEA** | **SRMR** | **TLI** | **CFI** | **AIC** | **BIC** |
| --- | --- | --- | --- | --- | --- | --- | --- | --- |
| *Fixed-effects analysis run on all studies (k= 8; N = 8,751)* | | | | | | | | |
| **1** | 6800.25 | 209 | 0.063 | 0.306 | 0.954 | 0.959 | 6382.25 | 4925.72 |
| **2** | 4222.56 | 194 | 0.051 | 0.155 | 0.970 | 0.975 | 3834.56 | 2482.57 |
| **3** | 4591.62 | 203 | 0.052 | 0.198 | 0.969 | 0.973 | 4185.62 | 2770.91 |
| **4** | 4373.22 | 202 | 0.051 | 0.167 | 0.970 | 0.974 | 3969.22 | 2561.48 |
| **5** | 4871.55 | 205 | 0.054 | 0.198 | 0.967 | 0.971 | 4461.55 | 3032.90 |
| **6** | 5854.37 | 205 | 0.059 | 0.222 | 0.960 | 0.964 | 5444.37 | 4015.72 |
| **7** | 6299.01 | 208 | 0.061 | 0.248 | 0.958 | 0.962 | 5883.01 | 4433.45 |
| **8** | **2904.79** | **188** | **0.043** | **0.107** | **0.979** | **0.983** | **2528.79** | **1218.61** |
| **9** | 3871.60 | 202 | 0.048 | 0.148 | 0.974 | 0.978 | 3467.60 | 2059.86 |

**Supplementary Table 18.** Model 2 (ITQ-22) factor loadings

|  | Re | Av | Th | Ad | Nsc | Dr |
| --- | --- | --- | --- | --- | --- | --- |
| Re | 1 |  |  |  |  |  |
| Av | 0.76 | 1 |  |  |  |  |
| Th | 0.77 | 0.80 | 1 |  |  |  |
| Ad | 0.68 | 0.74 | 0.77 | 1 |  |  |
| Nsc | 0.61 | 0.67 | 0.67 | 0.86 | 1 |  |
| Dr | 0.59 | 0.67 | 0.68 | 0.88 | 0.88 | 1 |
| *Note:* Ad, Affect dysregulation; Av; Avoidance; Dr, Disturbances in relationships; Nsc, Negative self-concept; Re, Re-experiencing; Th; Sense of threat. | | | | | | |

**Supplementary Table 19.** Model 8 (ITQ-22) factor loadings

|  | Re | Av | Th | Hyper | Hypo | Nsc | Dr | |
| --- | --- | --- | --- | --- | --- | --- | --- | --- |
| Re | 1 |  |  |  |  |  |  |  |
| Av | 0.75 | 1 |  |  |  |  |  |  |
| Th | 0.75 | 0.78 | 1 |  |  |  |  |  |
| Hyper | 0.56 | 0.63 | 0.69 | 1 |  |  |  |  |
| Hypo | 0.66 | 0.70 | 0.71 | 0.76 | 1 |  |  |  |
| Nsc | 0.58 | 0.64 | 0.63 | 0.70 | 0.84 | 1 |  |  |
| Dr | 0.56 | 0.64 | 0.63 | 0.69 | 0.89 | 0.88 | 1 |  |
| *Note:* Av; Avoidance; Dr, Disturbances in relationships; Hyper, Hyperactivation; Hypo, Hypoactivation; Nsc, Negative self-concept; Re, Re-experiencing; Th; Sense of threat. | | | | | | | |  |

**Supplementary Table 20.** Model fit statistics for modified ITQ measures

| **Model** | **χ2** | **DF** | **RMSEA** | **SRMR** | **TLI** | **CFI** | **AIC** | **BIC** |
| --- | --- | --- | --- | --- | --- | --- | --- | --- |
| *ITQ14a, fixed-effects analysis run on all studies (k = 8; N = 7,856)* | | | | | | | | |
| 2 | 1549.58 | 62 | 0.055 | 0.086 | 0.983 | 0.988 | 1425.58 | 993.49 |
| 4 | 1658.68 | 70 | 0.054 | 0.098 | 0.984 | 0.988 | 1518.68 | 1030.85 |
| **8** | **468.68** | **56** | **0.031** | **0.029** | **0.885** | **0.997** | **356.68** | **-33.58** |
| 9 | 860.23 | 69 | 0.038 | 0.073 | 0.992 | 0.994 | 722.23 | 241.37 |
| *ITQ14b, fixed-effects analysis run on all studies (k= 8; N = 7,856)* | | | | | | | | |
| 2 | 1819.02 | 62 | 0.060 | 0.093 | 0.980 | 0.987 | 1695.01 | 1262.94 |
| 4 | 1902.36 | 70 | 0.058 | 0.102 | 0.982 | 0.986 | 1762.36 | 1274.53 |
| **8** | **509.13** | **56** | **0.032** | **0.032** | **0.994** | **0.996** | **397.12** | **6.86** |
| 9 | 783.51 | 69 | 0.036 | 0.061 | 0.993 | 0.994 | 645.51 | 164.64 |
| *ITQ-20, fixed-effects analysis run on all studies (k= 8; N = 7,856)* | | | | | | | | |
| 2 | 3749.67 | 155 | 0.054 | 0.151 | 0.972 | 0.977 | 3439.67 | 2359.47 |
| 4 | 3899.02 | 163 | 0.054 | 0.164 | 0.972 | 0.976 | 3573.02 | 2437.07 |
| **8** | **2411.42** | **149** | **0.044** | **0.102** | **0.982** | **0.986** | **2113.43** | **1075.04** |
| 9 | 3259.40 | 163 | 0.049 | 0.141 | 0.977 | 0.980 | 2933.40 | 1797.45 |

**Supplementary Table 21.** Effects of study-level moderators on factor loadings

|  |  |  |  |  |  |  |
| --- | --- | --- | --- | --- | --- | --- |
| \| **Item** \| **Factor** \| **Factor Loading Change** \| **SE** \| **z value** \| **p value** \| \| --- \| --- \| --- \| --- \| --- \| --- \| \| *Diagnosed PTSD* \| \|  \|  \|  \|  \| \| Re1 \| Re \| -0.211 \| 0.024 \| -8.751 \| < .001 \| \| Re2 \| Re \| -0.196 \| 0.023 \| -8.607 \| < .001 \| \| Av1 \| Av \| -0.117 \| 0.022 \| -5.414 \| < .001 \| \| Av2 \| Av \| -0.101 \| 0.022 \| -4.52 \| < .001 \| \| Th1 \| Th \| -0.1 \| 0.025 \| -3.987 \| < .001 \| \| Th2 \| Th \| -0.147 \| 0.02 \| -7.281 \| < .001 \| \| Ad1 \| Ad \| -0.087 \| 0.021 \| -4.199 \| < .001 \| \| Ad2 \| Ad \| -0.113 \| 0.02 \| -5.654 \| < .001 \| \| Nsc1 \| Nsc \| -0.034 \| 0.018 \| -1.879 \| 0.06 \| \| Nsc2 \| Nsc \| -0.019 \| 0.018 \| -1.041 \| 0.298 \| \| Dr1 \| Dr \| -0.06 \| 0.017 \| -3.537 \| < .001 \| \| Dr2 \| Dr \| -0.116 \| 0.017 \| -6.649 \| < .001 \| \| *Probable CPTSD* \|  \|  \|  \|  \|  \| \| Re1 \| Re \| -0.186 \| 0.017 \| -11.147 \| < .001 \| \| Re2 \| Re \| -0.206 \| 0.016 \| -13.3 \| < .001 \| \| Av1 \| Av \| -0.173 \| 0.016 \| -10.599 \| < .001 \| \| Av2 \| Av \| -0.125 \| 0.016 \| -7.586 \| < .001 \| \| Th1 \| Th \| -0.122 \| 0.017 \| -7.071 \| < .001 \| \| Th2 \| Th \| -0.119 \| 0.015 \| -8.032 \| < .001 \| \| Ad1 \| Ad \| -0.062 \| 0.015 \| -4.042 \| < .001 \| \| Ad2 \| Ad \| -0.089 \| 0.015 \| -5.823 \| < .001 \| \| Nsc1 \| Nsc \| -0.044 \| 0.014 \| -3.044 \| 0.002 \| \| Nsc2 \| Nsc \| -0.024 \| 0.014 \| -1.674 \| 0.094 \| \| Dr1 \| Dr \| -0.056 \| 0.013 \| -4.253 \| < .001 \| \| Dr2 \| Dr \| -0.109 \| 0.015 \| -7.476 \| < .001 \| \| *CPTSD Severity* \|  \|  \|  \|  \|  \| \| Re1 \| Re \| -0.029 \| 0.008 \| -3.65 \| < .001 \| \| Re2 \| Re \| -0.036 \| 0.008 \| -4.706 \| < .001 \| \| Av1 \| Av \| -0.016 \| 0.008 \| -1.87 \| 0.061 \| \| Av2 \| Av \| -0.014 \| 0.008 \| -1.753 \| 0.08 \| \| Th1 \| Th \| -0.014 \| 0.008 \| -1.803 \| 0.071 \| \| Th2 \| Th \| -0.01 \| 0.008 \| -1.339 \| 0.181 \| \| Ad1 \| Ad \| 0.006 \| 0.007 \| 0.868 \| 0.385 \| \| Ad2 \| Ad \| -0.009 \| 0.007 \| -1.296 \| 0.195 \| \| Nsc1 \| Nsc \| -0.008 \| 0.006 \| -1.276 \| 0.202 \| \| Nsc2 \| Nsc \| 0.003 \| 0.006 \| 0.503 \| 0.615 \| \| Dr1 \| Dr \| 0.003 \| 0.006 \| 0.435 \| 0.664 \| \| Dr2 \| Dr \| -0.027 \| 0.007 \| -4.043 \| < .001 \| \| *English translation used* \|  \|  \|  \|  \|  \| \| Re1 \| Re \| 0.042 \| 0.015 \| 2.78 \| 0.005 \| \| Re2 \| Re \| 0.018 \| 0.015 \| 1.223 \| 0.221 \| \| Av1 \| Av \| 0.022 \| 0.012 \| 1.772 \| 0.076 \| \| Av2 \| Av \| 0.009 \| 0.013 \| 0.734 \| 0.463 \| \| Th1 \| Th \| -0.059 \| 0.014 \| -4.187 \| < .001 \| \| Th2 \| Th \| -0.023 \| 0.013 \| -1.724 \| 0.085 \| \| Ad1 \| Ad \| 0.036 \| 0.012 \| 2.992 \| 0.003 \| \| Ad2 \| Ad \| 0.041 \| 0.012 \| 3.428 \| 0.001 \| \| Nsc1 \| Nsc \| 0.018 \| 0.01 \| 1.725 \| 0.085 \| \| Nsc2 \| Nsc \| 0.026 \| 0.011 \| 2.463 \| 0.014 \| \| Dr1 \| Dr \| 0.015 \| 0.011 \| 1.45 \| 0.147 \| \| Dr2 \| Dr \| -0.012 \| 0.012 \| -1.045 \| 0.296 \| \| *Note:* Ad, Affect dysregulation; Av; Avoidance; Dr, Disturbances in relationships; Nsc, Negative self-concept; Re, Re-experiencing; Th; Sense of threat. \| \| \| \| \| \| | |  |  |  |  |  |

**Supplementary Table 22.** Reliability estimates for subgroups with the ITQ-12

| Model | Factor | PTSD sample (*k* = 9) | Non-PTSD sample (*k* = 48) | Probable CPTSD (*k* = 17) | Non-probable CPTSD (*k* = 41) | English (*k* = 19) | Translated (*k* = 39) |
| --- | --- | --- | --- | --- | --- | --- | --- |
| Model 2/Model 8 | Re | ω = 0.48 (.46-.50) | ω = 0.63 (.60-.66) | ω = 0.45 (.44-.47) | ω = 0.67 (.65-.69) | ω = 0.40 (.39-.42) | ω = 0.44 (.42-.45) |
|  | Av | ω = 0.61 (.60-.63) | ω = 0.71 (.68-.73) | ω = 0.60 (.59-.61) | ω = 0.73 (.71-.75) | ω = 0.51 (.50-.52) | ω = N/A |
|  | Th | ω = 0.60 (.59-.62) | ω = 0.67 (.64-.70) | ω = 0.58 (.57-.59) | ω = 0.69 (.66-.71) | ω = 0.40 (.40-.42) | ω = 0.48 (.47-.49) |
|  | Ad | ω = 0.35 (.33-.38) | ω = 0.45 (.43-.48) | ω = 0.37 (.35-.39) | ω = 0.47 (.44-.49) | ω = 0.31 (.29-.32) | ω = 0.21 (.20-.22) |
|  | Hyper | ω = N/A | ω = N/A | ω = N/A | ω = N/A | ω = N/A | ω = N/A |
|  | Hypo | ω = N/A | ω = N/A | ω = N/A | ω = N/A | ω = N/A | ω = N/A |
|  | Nsc | ω = 0.89 (.88-.90) | ω = 0.83 (.81-.85) | ω = 0.88 (.88-.89) | ω = 0.83 (.81-.85) | ω = 0.73 (.72-.74) | ω = 0.75 (.74-.75) |
|  | Dr | ω = 0.63 (.62-.65) | ω = 0.72 (.71-.74) | ω = 0.65 (.64-.67) | ω = 0.73 (.71-.74) | ω = 0.50 (.49-.52) | ω = 0.51 (.50-.52) |
| Model 4 | Core PTSD | ωho = 0.86 (.86-.86) | ωho = 0.85 (.85-.85) | ωho = 0.74 (.73-.75) | ωho = 0.86 (.86-.86) | ωho = 0.86 (.86-.87) | ωho = 0.86 (.85-.86) |
|  | DSO | ωho = 0.88 (.87-.88) | ωho = 0.87 (.87-.88) | ωho = 0.84 (.83-.84) | ωho = 0.88 (.87-.88) | ωho = 0.89 (.89-.90) | ωho = 0.88 (.88-.88) |
| *Note:* Ad, Affect dysregulation; Av; Avoidance; Dr, Disturbances in relationships; Nsc, Negative self-concept; Re, Re experiencing; Th; Sense of threat. | | | | | | | |

|  |  |  |  |  |  |  |
| --- | --- | --- | --- | --- | --- | --- |
|  |  |  |  |  |  |  |
|  |  |  |  |  |  |  |
|  |  |  |  |  |  |  |
|  |  |  |  |  |  |  |
|  |  |  |  |  |  |  |
